# Quantitative Protein Localization Signatures Reveal an Association between Spatial and Functional Divergences of Proteins

**DOI:** 10.1371/journal.pcbi.1003504

**Published:** 2014-03-06

**Authors:** Lit-Hsin Loo, Danai Laksameethanasan, Yi-Ling Tung

**Affiliations:** 1 Bioinformatics Institute, Agency for Science, Technology and Research, Singapore, Singapore; 2 Department of Pharmacology, Yong Loo Lin School of Medicine, National University of Singapore, Singapore, Singapore; University of Zurich and Swiss Institute of Bioinformatics, Switzerland

## Abstract

Protein subcellular localization is a major determinant of protein function. However, this important protein feature is often described in terms of discrete and qualitative categories of subcellular compartments, and therefore it has limited applications in quantitative protein function analyses. Here, we present Protein Localization Analysis and Search Tools (PLAST), an automated analysis framework for constructing and comparing quantitative signatures of protein subcellular localization patterns based on microscopy images. PLAST produces human-interpretable protein localization maps that quantitatively describe the similarities in the localization patterns of proteins and major subcellular compartments, without requiring manual assignment or supervised learning of these compartments. Using the budding yeast *Saccharomyces cerevisiae* as a model system, we show that PLAST is more accurate than existing, qualitative protein localization annotations in identifying known co-localized proteins. Furthermore, we demonstrate that PLAST can reveal protein localization-function relationships that are not obvious from these annotations. First, we identified proteins that have similar localization patterns and participate in closely-related biological processes, but do not necessarily form stable complexes with each other or localize at the same organelles. Second, we found an association between spatial and functional divergences of proteins during evolution. Surprisingly, as proteins with common ancestors evolve, they tend to develop more diverged subcellular localization patterns, but still occupy similar numbers of compartments. This suggests that divergence of protein localization might be more frequently due to the development of more specific localization patterns over ancestral compartments than the occupation of new compartments. PLAST enables systematic and quantitative analyses of protein localization-function relationships, and will be useful to elucidate protein functions and how these functions were acquired in cells from different organisms or species. A public web interface of PLAST is available at http://plast.bii.a-star.edu.sg.

## Author Summary

Proteins are fundamental building blocks of cells. They perform a variety of biological functions, many of which are essential to the vitality and normal functioning of cells. Proteins have to be located at the appropriate regions inside a cell to perform their functions. Therefore, when proteins change their locations, they may acquire new or different functions. However, the relationships between the locations and functions of proteins are difficult to analyze because protein locations are often represented in distinct and manually-defined categories of subcellular regions. Many proteins have complex or subtle differences in their localization patterns that can be hardly represented by these categories. Here, we present an automated analysis tool for generating quantitative signatures of protein localization patterns without requiring manual or automated assignments of proteins into distinct categories. We show that our tool can identify proteins located at the same subcellular regions more accurately than existing categorization-based methods. Our tool allows comprehensive and more accurate studies of the relationships between protein localizations and functions. By knowing where proteins are located and how their locations were changed, we may discover their functions and better understand how they acquire these functions.

## Introduction

Proteins have to be localized at the appropriate subcellular compartments to perform their functions. Using high-throughput protein labeling and imaging techniques, several proteome-wide studies have found that protein localization and function are strongly correlated to each other [1]–[4]. However, unlike sequence, structure, expression level, or other protein features, subcellular localization has limited applications in quantitative analyses of protein functions, such as predictions of protein functions [5] and studies of protein function evolution [6], [7].

One of the main difficulties is that protein subcellular localization is often described or represented in terms of discrete, qualitative categories of subcellular compartments, such as the Gene Ontology (GO) categories [8]. Although automated image processing algorithms have been useful in extracting quantitative descriptors for protein localization patterns, the resulting descriptors are often being converted back into these discrete categories using supervised classification or unsupervised clustering methods [9]–[13]. These discrete representations have several limitations. First, they cannot fully describe the continuous and complex spatial distributions of proteins that are localized across multiple compartments [14] and/or distribute non-uniformly within the same compartments [15]. Second, they are often assigned based on manual and/or visual inspections [1], [3], [4], which are prone to bias and imprecision. Third, they only allow simple qualitative comparisons of protein localization patterns, which are often insufficient to distinguish complex or subtle changes. Despite all these limitations, discrete categories of subcellular compartments are still commonly used because they can be easily interpreted by humans.

To overcome these limitations, we have developed an automated analysis framework for converting raw image descriptors into quantitative signatures (or “profiles”) of protein subcellular localization patterns. We refer to this framework as Protein Localization Analysis and Search Tools (PLAST). First, we measure a large number of unbiased image descriptors that capture different spatial properties of protein localization patterns, and use a support vector machine (SVM) algorithm [16] to reduce the contributions of non-informative descriptors. The resulting profiles allow PLAST to maintain continuous representations of protein localization patterns throughout its analysis workflow, and do not require supervised learning of pre- or manually-defined categories of subcellular compartments. Second, PLAST is fully automated and designed to systematically quantify protein localization patterns at the proteome scale. Third, PLAST allows quantitative comparisons of complex protein localization patterns based on standard dissimilarity or distance measurements. Last, PLAST produces human-interpretable protein localization maps that quantitatively describe the similarities in the localization patterns of proteins and major subcellular compartments. These maps can be thresholded to make “hard” compartment-to-protein assignments at user-desired significance levels. Therefore, PLAST allows researchers to quantify and rank a set of proteins according to their localization dissimilarities to a given protein or organelle, much like searching for proteins with similar sequences in the GenBank or UniProt databases.

An important application of PLAST is to study changes in protein localization and function during evolution. Gene duplication is a main source of new genes [6], [17]. A fundamental question in evolutionary biology is how duplicate genes acquired new or altered biological functions. Change in protein subcellular localization, or “protein relocalization”, is a possible mechanism for duplicate genes to achieve functional divergence [18]. This is supported by observations that protein localization may be easily changed just by single amino-acid substitutions [18], relocalization is sufficient to alter the functions of some enzymes even in the absence of any mutation in their catalytic sites [19], and many gene families encode proteins with different subcellular localizations [20], [21]. Two different models of protein relocalization have been proposed [22]. The “neolocalization” model suggests that duplicates relocalize and adapt to previously unoccupied compartments [23], whereas the “sublocalization” model suggests that duplicates develop more specific localization patterns over their ancestral compartments [24]. Based on these models, neolocalized duplicates are expected to occupy higher total numbers of compartments than their ancestors; and conversely, sublocalized duplicates are expected to occupy similar total numbers of compartments as their ancestors. However, both neo- and sublocalized duplicates are expected to show more diverged subcellular localization patterns and occupy lower ratios of shared compartments as they evolve. PLAST allows us to compare and test these two models by quantitatively measuring the degree of spatial divergence between duplicates and the number of compartments occupied and shared by them.

Here, we describe the key components of PLAST, and show that PLAST is, on average, more accurate than existing, qualitative protein localization annotations in identifying known co-localized proteins. Furthermore, we demonstrate that PLAST can reveal protein localization-function relationships that are not obvious from these annotations. We found that PLAST can 1) identify similarly-localized proteins that participate in closely-related biological processes but do not necessary form stable complexes with each other or localize at the same organelles, and 2) reveal an association between spatial and functional divergences of proteins during evolution.

## Results

### Overview of PLAST

**Figure 1:**
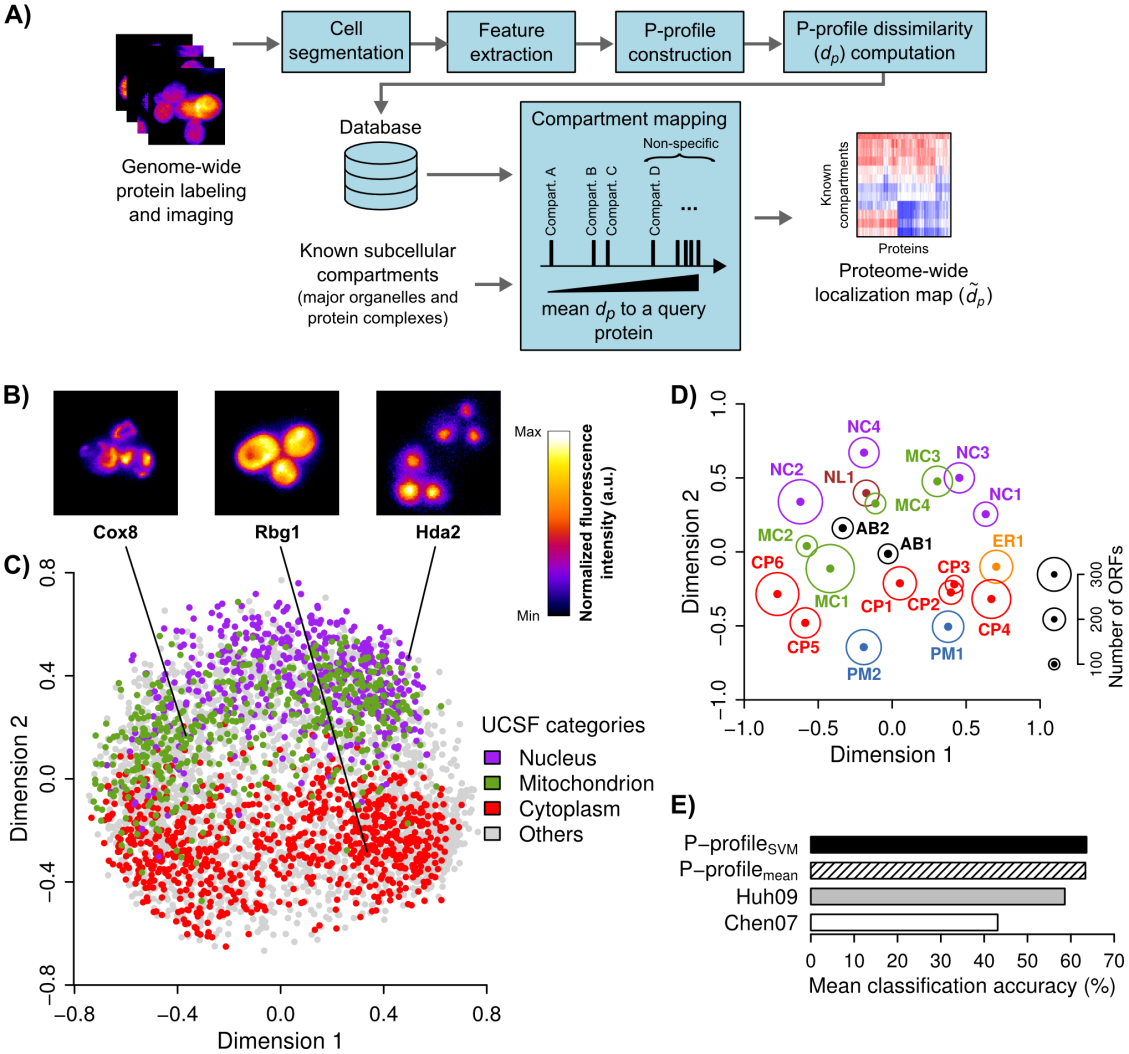
Construction of quantitative protein subcellular localization profiles. (**A**) Schematic showing the major components of Protein Localization Analysis and Search Tools (PLAST). (**B**) Example images of GFP-tagged *Saccharomyces cerevisiae* strains from the UCSF dataset [1]. The intensity of each image has been scaled to the same range. (**C**) Multi-dimensional scaling plot based on the dissimilarity scores (*d_p_*) among all the P-profiles_SVM_ constructed for the UCSF dataset. ORFs manually assigned to “nucleus”, “cytoplasm”, or “mitochondrion” categories by UCSF are shown in purple, red, or green dots, respectively. (**D**) Multidimensional scaling plot of 20 representative protein localization patterns (dots) or “exemplars” identified using an affinity-propagation clustering algorithm. The radius of the circle around each dot is proportional to the number of ORFs assigned to the exemplar. Each exemplar is colored and named according to the most enriched UCSF category among its assigned ORFs (**Supplementary Fig. S4A**). The exemplars of MC2 (Cox8), CP3 (Rbg1), and NC3 (Hda2) are shown in B. (**E**) Comparison of the performances of P-profiles and quantitative features extracted using two other previous analysis frameworks (“Chen07” and “Huh09”) [11], [13] in classifying ORFs according to UCSF categories. The accuracies shown were estimated using a multi-class SVM classifier and 5-fold cross validation, and averaged over all UCSF categories.

PLAST can be generally applied to microscopy images of proteins labeled with fluorescent protein fusion tags, fluorophore-conjugated antibodies, or other labeling techniques. PLAST has five major steps: cell segmentation, feature extraction, protein localization profile (“P-profile”) construction, P-profile dissimilarity computation, and compartment mapping (**Fig. 1A**). First, we automatically segment cells from microscopy images. To avoid segmentation bias that may be introduced by protein-to-protein variations in expression levels [25], we do not use fluorescent signals from the labeled proteins. Instead, we have developed a segmentation algorithm based on differential interference contrast (DIC) illumination and fluorescent nuclear stains (**Supplementary Fig. S1**). Other segmentation algorithms based on fluorescent whole-cell stains [26] may also be used in this step.

Second, we extract a large number of quantitative image descriptors (or “features”) from the segmented cells. We have designed several new image features based on subcellular regions and local structures of protein distribution patterns (**Local structure features**). We also extract standard intensity, texture, and moment features [9], [10], [27].

Third, we have generalized a SVM-based drug profiling algorithm [27], which was originally designed to compare the same proteins under perturbed and control conditions, to compare different proteins under the same cellular conditions. For each protein, this method removes the contributions of non-informative features by finding an optimum SVM hyperplane that can separate cells labeled for the protein from a fixed set of reference cells (**P-profile_SVM_ construction** and **Fig. S2A**). We used the unit vector orthogonal to the hyperplane as a quantitative profile representing the spatial localization signature of the protein. As an alternative, we also construct profiles by averaging each feature value across all cells. We denote these two profile types as “P-profile_SVM_” and “P-profile_mean_”, respectively.

Fourth, we measure and store the pairwise dissimilarity scores (*d_p_*) between the P-profiles of all proteins into a database. Lower *d_p_* values correspond to more similar subcellular localization patterns. To compare the localization patterns of a protein to a group of proteins, such as those that constitute a subcellular organelle or compartment, we compute the mean of all the pairwise *d_p_* values between the protein and each of the group members (**P-profile dissimilarity score**). If the protein is part of the group, the *d_p_* to itself will be excluded from the calculation.

Fifth, we map a comprehensive catalog of major subcellular compartments to each of the proteins in our database. For each protein, we estimate the probability distribution of *d_p_* between the protein and the compartments that are not specifically occupied by the protein (**Fig. 1A**). Then, we standardize the *d_p_* values between the protein and all compartments based on this distribution (**Compartment assignment**). The resulting z-scores (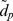) constitute a localization map of all the proteins, and allow us to use the same significance thresholds to assign compartments to proteins.

### Subcellular localization profiles of the yeast proteome

To assess the performance of PLAST, we used the budding yeast *Saccharomyces cerevisiae* (*S. cerevisiae*) as a model system due to the availability of a genome-wide GFP-fusion-protein image dataset (**Fig. 1B**, the “UCSF dataset”) [1] and a large number of known protein complexes in this organism [28]. The dataset covers ∼75% of the *S. cerevisiae* proteome. We extracted 623 quantitative features from ∼20 single cells per yeast strain, and constructed P-profiles for all the strains (**Supplementary Fig. S2A** and **B**). After quality control (**Quality control**), we obtained P-profiles for 4066 open reading frames (ORFs) (**Fig. 1C**).

To determine the correspondence between P-profiles and compartment categories manually assigned by UCSF via visual inspections (“UCSF categories”), we clustered P-profiles using an affinity propagation algorithm [29] (**Fig. 1D** and **Supplementary Fig. S3**) and determined the enrichments of UCSF categories and GO biological processes in each of the 20 identified clusters. We found that PLAST divides most “cytoplasmic”, “nuclear”, or “mitochondrial” proteins into ∼4 to 6 clusters that are enriched in proteins involved in different biological processes (**Supplementary Fig. S4**). Thus, PLAST can reveal protein subcellular localization patterns that are not obvious under manual inspections.

Previously, two other analysis frameworks were also developed to quantify and classify the same image dataset according to UCSF categories [11], [13]. We found that P-profiles can achieve higher mean classification accuracy than these two previous frameworks (**Fig. 1E**). The increase in accuracy mostly came from better detections of categories with small numbers of ORFs, such as “early Golgi”, “bud neck” and “microtubule”, which were completely missed by these two other frameworks (**Supplementary Fig. S2C**). These improvements were likely due to our better cell segmentation algorithm that does not rely on the fluorescence intensities of the GFP-tagged proteins (**Supplementary Fig. S1**), and our SVM-based profiling algorithm that reduces the contributions of non-informative features.

### Performance in identifying co-localized proteins

**Figure 2:**
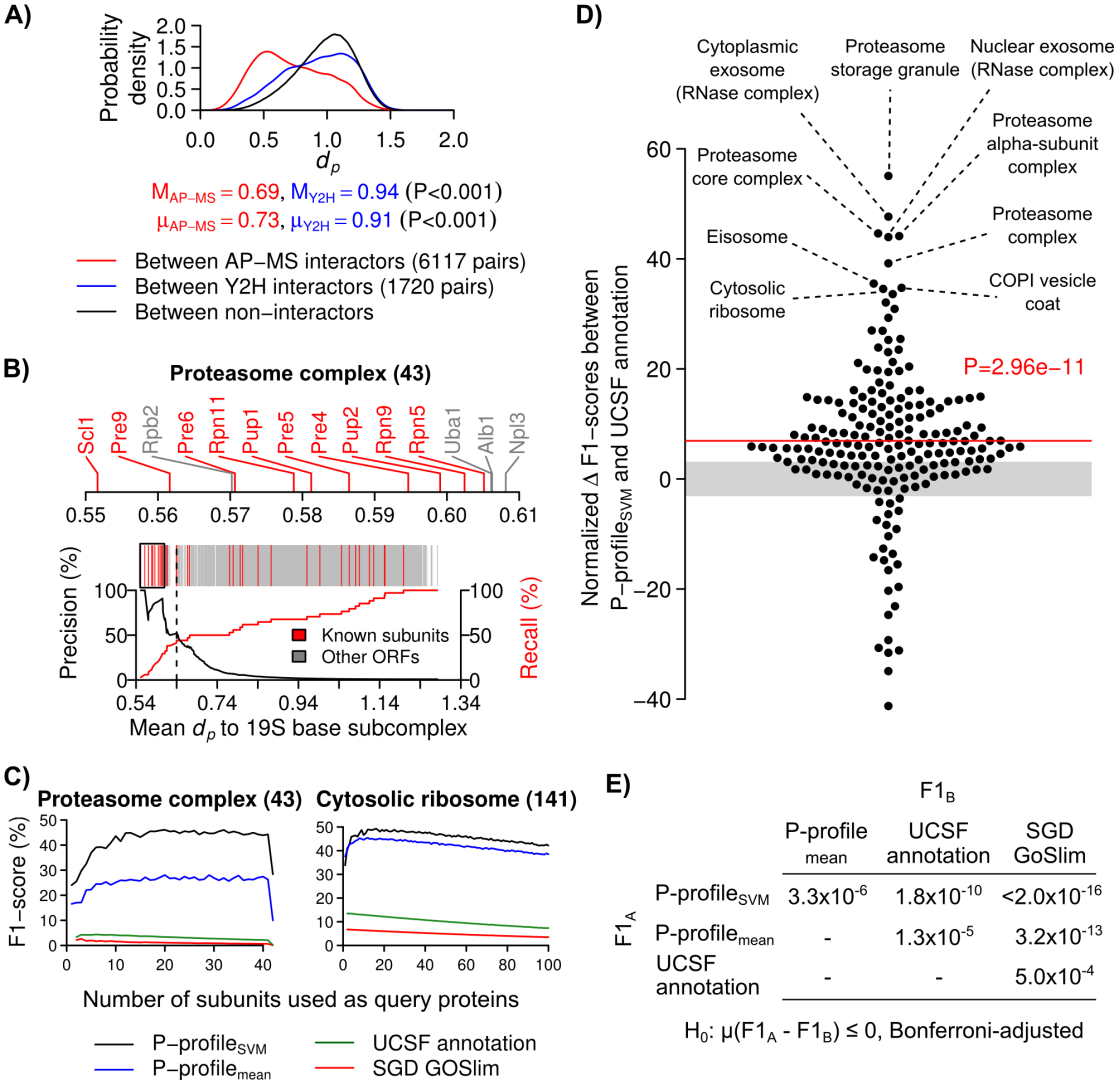
Performance of PLAST in identifying known co-localized proteins. (**A**) Probability distributions of the P-profile dissimilarity scores (*d_p_*) between interactors and between non-interactors detected by affinity-purification mass spectrometry (AP-MS) or yeast two-hybrid screening (Y2H). (M = medians, μ = means of the distributions; P-values from two-sided permutation tests for differences in means or medians.) (**B**) An example of PLAST search result obtained from using 19S proteasomal base subunits as query proteins. The mean *d_p_* between the query proteins and all other proteins are shown as red (known subunits) or gray (other ORFs) vertical lines. Most of the red lines have low *d_p_* values, indicating that they are placed at the top of the search result. (Black line graph = precisions, red line graph = recalls, black dashed line = decision threshold at optimum F1-score, black box = magnified region, parenthesis = number of known subunits.) (**C**) Performances of subunit searches obtained from using different numbers of query proteins randomly selected from known subunits of a proteasome (left) or cytosolic ribosome (right). For each query protein number, we tested max(100, number of all possible combinations) random combinations of query proteins, and computed the mean value of these tested combinations (parentheses = numbers of known subunits). (**D**) Normalized F1-score differences between P-profile_SVM_ and UCSF annotation for a catalog of 197 protein complexes. Some of the complexes with the highest F1-score differences are highlighted. (Red line = the mean of the normalized differences, which is also the test statistic used in the paired t-test between the F1-scores of these two methods; gray areas = statistically insignificant differences with P>0.001; red text = unadjusted P-value obtained for the paired t-test.) (**E**) Bonferroni-adjusted P-values obtained from one-sided, paired t-tests between the F1-scores of of all the possible pairs of profiling/annotation methods (F1_A_ vs. F1_B_).

Proteins must localize in close proximity to interact physically. We next studied to what extent PLAST can be used to search for physically interacting proteins. We first made use of two high-quality protein-protein interaction datasets obtained from affinity-purification mass spectrometry (AP-MS) and yeast two-hybrid (Y2H) screening [30]. AP-MS can identify components of larger complexes that may not necessary directly interact with each other, whereas Y2H screening can identify direct and sometimes more transient interactions between components from different complexes or pathways [30]. We found that AP-MS interactors have significantly lower median and mean intrapair *d_p_* values than Y2H interactors (P<0.001, two-sided permutation test; **Fig. 2A**). These results show that PLAST is better in detecting stable protein complexes than transient interactors.

To further test the ability of PLAST in searching subunits of stable protein complexes, we used a comprehensive catalog of 197 protein complexes with different subunit numbers and subcellular localization patterns (**Datasets**). Identifying subunits of complexes that localized at multiple compartments, such as ribosome and proteasome [15], [31], is expected to be very challenging. For each complex, we randomly selected a subset of its subunits as query proteins, and ranked all other proteins, except the query proteins, according to their mean *d_p_* to the query proteins (**Fig. 2B**). We systematically measured the precision, recall and F1 scores, which are commonly-used criteria for information retrieval performance [32], for different numbers of query proteins (**Fig. 2C**). We also ranked proteins based on their similarities in UCSF or SGD GoSlim localization annotations (**Subunit search based on UCSF or SGD GOSlim annotations**). These two annotations are not independent from each other, because a large fraction of SGD GoSlim annotations are based on UCSF annotations. We performed paired t-tests between the maximum F1 scores obtained from different profiling/annotation methods for all the protein complexes (**Fig. 2D and E**). The resulting test statistic is only weakly correlated to protein complex size (R = −0.136, P = 0.056; **Fig. S5**). Overall, we found that predictions based on P-profiles_SVM_ have significantly higher maximum F1 scores than other methods (P<3.3×10^−6^, Bonferroni-adjusted, one-sided paired t-test; **Figs. 2C and E**). Therefore, in the subsequent analyses, we will be using P-profiles_SVM_ to represent protein subcellular localization patterns. Notably, some of the “false positives” selected by PLAST may also interact with the query proteins. For example, RNA polymerase II subunit (Rpb2) and ubiquitin activating enzyme (Uba1) were previously found to be physically associated with proteasome [33], [34] (**Fig. 2B**). Thus, our estimated performances of PLAST are conservative. Our results show that, at least for most of the tested protein complexes, P-profiles are more accurate than existing localization annotations in associating subunits of the same protein complexes together.

### Construction of a subcellular localization map

**Figure 3:**
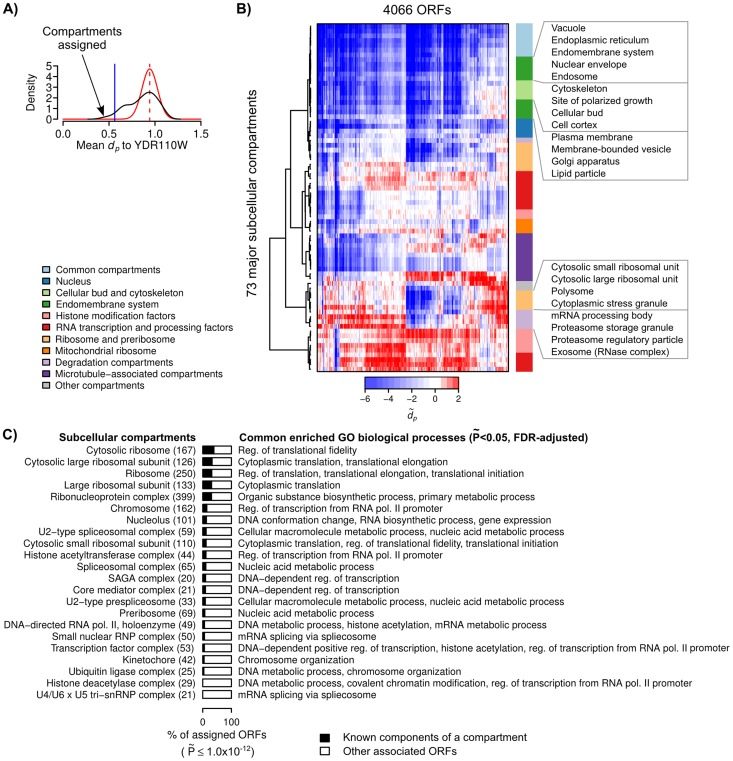
A subcellular localization map for the yeast proteome. (**A**) An example of how PLAST assigns compartments to an ORF, YDR110W (black curve = estimated probability distribution of the *d_p_* scores between the ORF and a catalog of 73 major subcellular compartments; dashed red vertical line = local maxima of the distribution with the highest *d_p_* value; red curve = estimated “null” distribution of the *d_p_* scores between the ORF and non-specifically localized compartments; blue vertical line = a threshold for compartments with *d_p_* significantly less than the null distribution at Bonferroni-adjusted P˜<2.5×10^−4^.) The estimated mean and standard deviation of the null distribution are used to standardize the *d_p_* scores between the ORF and all compartments. (**B**) A subcellular localization map showing the standardized P-profile dissimilariy scores (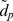) between 4066 ORFs (x-axis) and the 73 major subcellular compartments (y-axis) in a budding yeast cell. The compartments (rows) were ordered using a hierarchical clustering algorithm with cosine dissimilarity scores, and labeled with color codes according to their known functions or localizations (“common” compartments = compartments assigned to large numbers of ORFs.) A fully annotated map is shown in **Supplementary Fig. S7**. (**C**) Using a Bonferroni-adjusted threshold of P˜<1.0×10^−12^, we assigned compartments to each and every ORF. Among the 73 compartments, we found 22 compartments whose known components and “non-components” assigned by PLAST share at least one common, significantly-enriched GO biological process (P˜<0.05 with false-discovery-rate adjustment, hypergeometric test). Shown are the percentages of known- and non-components in all the ORFs assigned with these compartments by PLAST. The list of (up to three) common enriched GO biological processes for each compartment is also shown (pol. = polymerase, reg. = regulation, RNP = ribonucleoprotein).

To generate a human-interpretable localization map of the yeast proteome, we used another catalog of cellular compartments as “landmarks” of the subcellular space in a yeast cell. This catalog consists of known protein components of 23 major organelles and 50 large protein complexes (**Catalog of subcellular compartments**). For each protein, we systematically queried the P-profile database for the *d_p_* scores between the protein and all the compartments. We assumed that the probability distribution for these *d_p_* scores could be modeled by a mixture of Gaussian distributions, in which the component distribution with the highest mean *d_p_* value was the distribution for non-specifically localized compartments. We estimated the mean and standard deviation of this “null” distribution and standardized all the *d_p_* scores based on the estimated values (**Compartment assignment** and **Fig. 3A**). We found that the null distributions for most proteins are dominant (**Supplementary Fig. S6**), indicating most proteins are localized only at small subsets of components in our catalog. The resulting z-scores (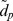) constitute the final localization map (**Fig. 3B** **and S7**, **Supplementary Dataset S1**), and allow us to assign compartments to different proteins using the same Bonferroni-adjusted P-value thresholds. This standardization step does not change the relative dissimilarities of different compartments to a protein.

### Spatially-associated proteins tend to participate in related biological processes

We identified several general trends from this localization map. First, compartments involved in similar or related biological processes tend to be spatially associated to similar sets of proteins. We performed a hierarchical clustering of all the compartments based on the localization map, and found that functionally-related compartments tend to be grouped into the same clusters (**Fig. 3B** **and S7**). For example, we obtained two clusters of compartments that are related to cellular bud/cytoskeleton and endomembrane system, respectively. These two clusters are also closest to each other, and form a larger supercluster in the dendrogram (**Fig. 3B**). Similarly, we obtained separate clusters for ribosomal subunits, polysome, and degradation compartments, which together also form a larger supercluster (**Fig. 3B**). These protein machineries are known to work together to regulate gene expression [35]. The spatial associations of these functionally-related compartments suggest that PLAST, which is based on microscopy images, can identify spatially-associated proteins that participate in closely-related biological processes or pathways but do not necessary form stable complexes with each other or localize at the same organelles. Most affinity-purification-based methods, such as the aforementioned AP-MS method, would have difficulties in relating these proteins together.

To perform hard assignments of compartments to proteins, we used a Bonferroni-adjusted threshold of 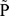
<1.0×10^−12^. We studied all the compartments that had been assigned to at least one protein, and found that, on average, ∼90% of the proteins assigned to a compartment are not known components of the compartment. We wonder to what extent these “non-components” may perform similar biological functions as other known components of the compartment. For each compartment, we systematically identified significantly enriched GO biological processes among all its known components, and among all its non-components assigned by PLAST. Interestingly, we found 22 compartments whose non- and known components are significantly enriched with at least one common biological process (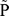
<0.05, FDR-adjusted, hypergeometric test; **Fig. 3C** and **Supplementary Dataset S2**). For example, several translation factors (eIF1A, eIF4B, and eIF4G) and synthases (glycyl-tRNA and glutamyl-tRNA synthases) were assigned to cytosolic ribosomes; and several splicing factors (Prp11, Prp39, Prp45, and Prp5), topoisomerase (Top2), and transcription initiation factor (Tfc8) were assigned to RNA polymerase II (**Supplementary Dataset S2**). Furthermore, we also found that the non-components assigned by PLAST to cytosolic ribosome were also significantly enriched with ORFs that were experimentally found to co-purify with cytosolic ribosome [36] (P<0.001, hypergeometric test; **Fig. S8**). Importantly, some of these non-components have uncharacterized functions or not known to be functionally associated with their corresponding compartments. Therefore, our localization map provides a repertoire of potentially novel components of the biological processes or pathways performed at major subcellular compartments in the budding yeast.

### Most proteins are localized at multiple compartments

**Figure 4:**
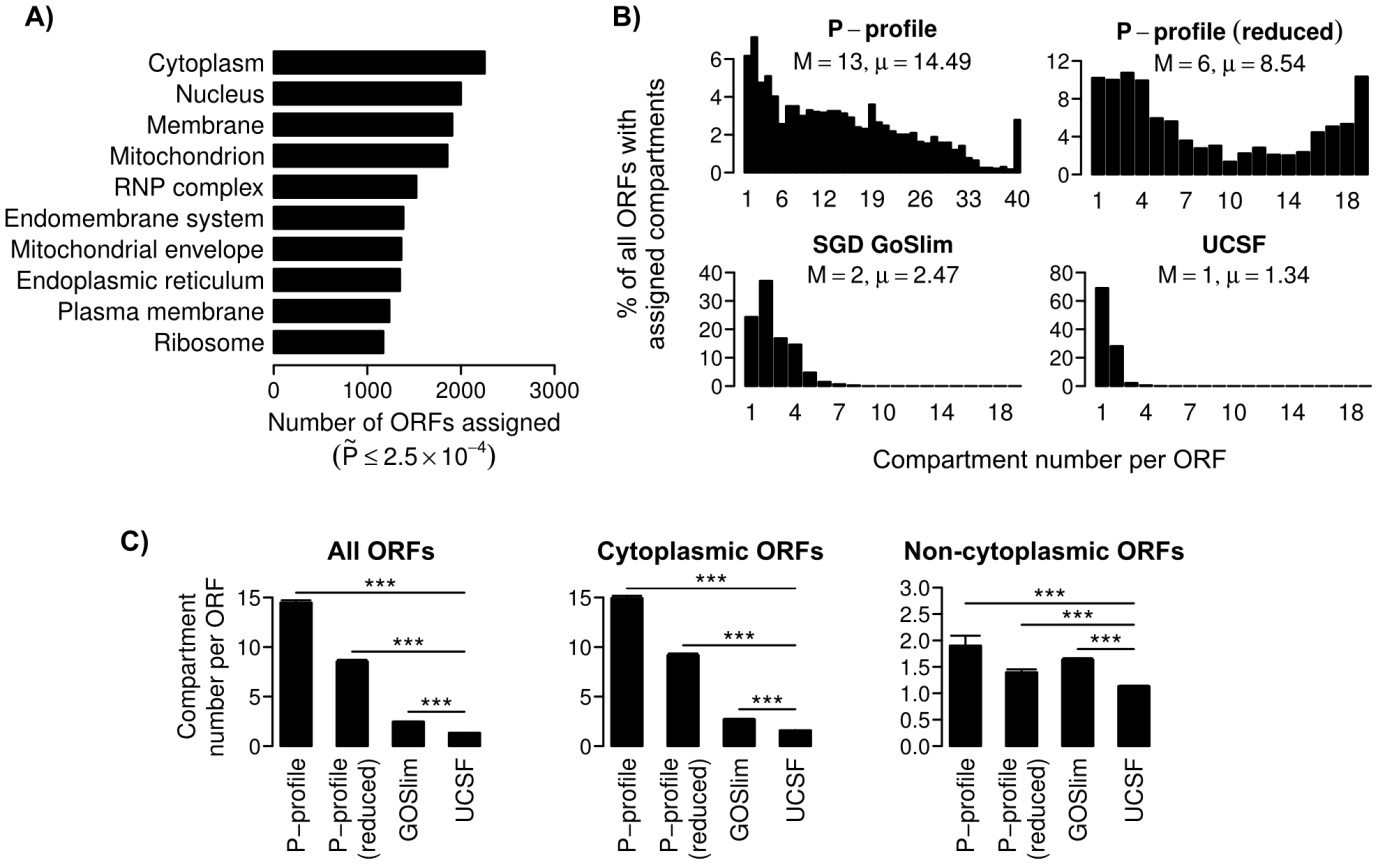
Most proteins are localized at multiple subcellular compartments. (**A**) The ten compartments with the highest numbers of assigned ORFs at a Bonferroni-adjusted threshold of 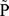
<2.5×10^−4^ (RNP = ribonucleoprotein). (**B**) Distributions of ORFs with different numbers of assigned subcellular compartments. The assignments were based on P-profile_SVM_ with all 73 compartments, P-profile_SVM_ with a reduced set of 22 compartments, UCSF, and SGD GoSlim cellular component annotations (M = medians, μ = means of the distributions). (**C**) Comparisons of the mean numbers of compartments assigned to an ORF by different profiling/annotation methods. (Cytoplasmic/Non-cytoplasmic ORFs = ORFs assigned or not assigned with cytoplasm, respectively; error bars = standard errors; *** = P<0.001, two-sided permutation test for the difference in means.)

The second general trend is that most proteins are localized at multiple compartments. To perform a fair comparison between PLAST and UCSF annotations, we used a more relaxed Bonferroni-adjusted threshold of 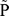
≤2.5×10^−4^, which assigns major organelles, namely cytoplasm and nucleus, to similar numbers of proteins as UCSF annotations [1] (**Fig. 4A**). However, among proteins with assigned compartments, the median and mean numbers of compartments assigned to a protein by PLAST (∼13.0 to 14.5) are significantly higher than UCSF (∼1) and SGD GOSlim annotations (∼2.0 to 2.5, **Fig. 4B**). We observed the same trends even if we used a reduced set of 22 major compartments for compartment mapping by PLAST (**Fig. 4B**). Many of the compartments that we used as landmarks are located in the cytoplasm, therefore it is not surprising that PLAST would also assign them to proteins that are localized at the cytoplasm. On average, PLAST assigned 14.9 compartments to cytosolic proteins, but only 1.9 compartments to non-cytosolic proteins (**Fig. 4C**). Nevertheless, in all cases, PLAST and SGD GoSlim assign significantly more compartments than UCSF annotations (P<0.001, **Fig. 4C**). This is likely due to the inefficiency of human scorers to separate complex composite patterns into individual compartments, and therefore analyses of protein localization based on UCSF annotations may be inaccurate.

### Duplicates with different divergence times occupy similar numbers of compartments

An important application of PLAST is to study spatial and functional divergences of proteins during evolution. Individual cases of neo- and sublocalization (**Introduction**) have been reported in yeasts and hominoids [22]–[24], and thus both mechanisms were likely to contribute to protein relocalization. Given that the subcellular localization data for ancestral proteins is not available, we use two different approaches to test the prevalence of neo- and sublocalization models. All our subsequent analyses are based on the compartment assignments from the previous section, and also P-profile_SVM_, which has the best overall benchmark performance (**Fig. 2E**).

**Figure 5:**
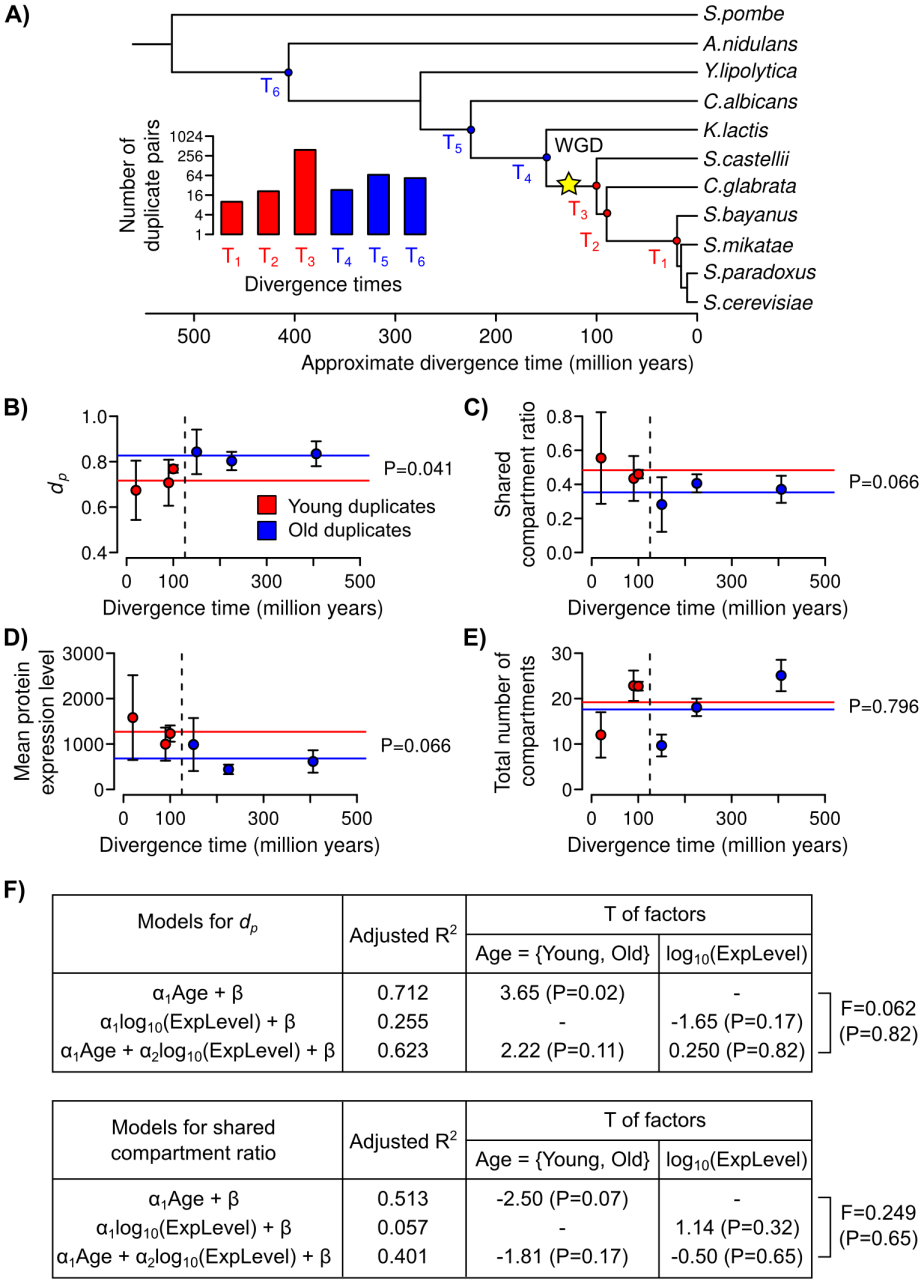
Duplicates with different divergence times have significantly different spatial divergence levels but similar numbers of occupied subcellular compartments. (**A**) We used a phylogeny of orhtologous gene groups estimated for Ascomycota fungi [37] (inset = number of *S. cerevisiae* duplicates that we could traced to their originating ancestors without any loss event; blue = “old” duplicates; red = “young” duplicates; WGD = whole genome duplication.) The (**B**) mean P-profile dissimilarity scores (*d_p_*), (**C**) shared compartment ratios, (**D**) mean protein expression levels, and (**E**) total numbers of occupied compartments for duplicates with different divergence times (dashed black vertical line = division between “young” and “old” duplicates; error bars = standard errors; red/blue line = mean values for the young or old duplicates, respectively; P-values from two-sided t-tests between young and old duplicates.) (**F**) Results from linear regression modeling of *d_p_* and shared compartment ratio using divergence age and protein expression level as factors (T = T-statistics of the factors; F = F-statistics for the analyses of variance (ANOVA) between two regression models with different factors; R^2^ = squared correlation coefficients between the actual and predicted values of *d_p_* or shared compartment ratio.)

In the first approach, we studied duplicates with different divergence times. We assumed that the localization patterns of more recently duplicated gene pairs are more similar to their ancestors. We used a phylogeny of orthologous gene groups estimated for seventeen Ascomycota fungi [37]. We obtained six sets of *S. cerevisiae* duplicates with different divergence times (T_1_ to T_6_), each of which consists of ∼10–400 duplicate pairs (**Fig. 5A**). We used PLAST to quantify the spatial divergence levels of all the duplicates. Because protein expression may influence protein localization pattern, we also obtained the protein expression levels of all the duplicates [25]. To treat each divergence time equally, we only used the mean values of all its associated duplicates. A whole genome duplication (WGD) was estimated to occur around ∼100 million years ago after the divergence of *Kluyveromyces lactis* (*K. lactis*) in the phylogeny [6], [38] (**Fig. 5A**). We refer to pre-WGD duplicates as “old” duplicates (T_4_ to T_6_), and all other duplicates as “young” duplicates (T_1_ to T_3_). We found that old duplicates have significantly larger intrapair *d_p_* values, lower shared compartment ratios, and lower average protein expression levels than young duplicates (P = 0.041, 0.066, and 0.066, two-sided t-tests; **Fig. 5B–D**). However, we did not observe a significant difference in the total numbers of occupied compartments (P = 0.796, two-sided t-test; **Fig. 5E**). To test if localization divergence could be predicted based on divergence time or protein expression level, we performed linear regression modeling and found that only divergence time is a significant predictor of both *d_p_* and shared compartment ratio (P<0.001, **Fig. 5F**). Our results are consistent with the expected behaviors of sublocalized duplicates (**Introductions**), and suggest that localization divergence might be more frequently due to sublocalization. One of the limitations of our analysis is that duplicates with different divergence times may undergo different evolutionary paths. Furthermore, we could only obtain small numbers (∼10) of duplicates for some of the divergence times, and thus could not perform meaningful enrichment analyses of protein functions on them.

### Large portion of WGD duplicate proteins have diverged localization patterns

In the second approach, we addressed these limitations by studying the large number (∼544) of duplicates generated from WGD [38], [39]. We assumed that the localization patterns of duplicates with lower intrapair *d_p_* values are more similar to their ancestors. Sixty eight percent of these WGD duplicates were detected in T_3_ of the phylogeny that we used in the previous section. Based on UCSF annotations, a previous study estimated that ∼24–37% of these WGD duplicates now have diverged localizations and occupy significantly higher numbers of compartments than their ancestors [22]. Thus, the study concluded that localization divergence might be more frequently due to neolocalization. However, UCSF annotations tend to underestimate the number of compartments localized by a protein (**Fig. 4C**), and we wonder if PLAST can provide additional insights into the localization-function relationships between these duplicate proteins.

**Figure 6:**
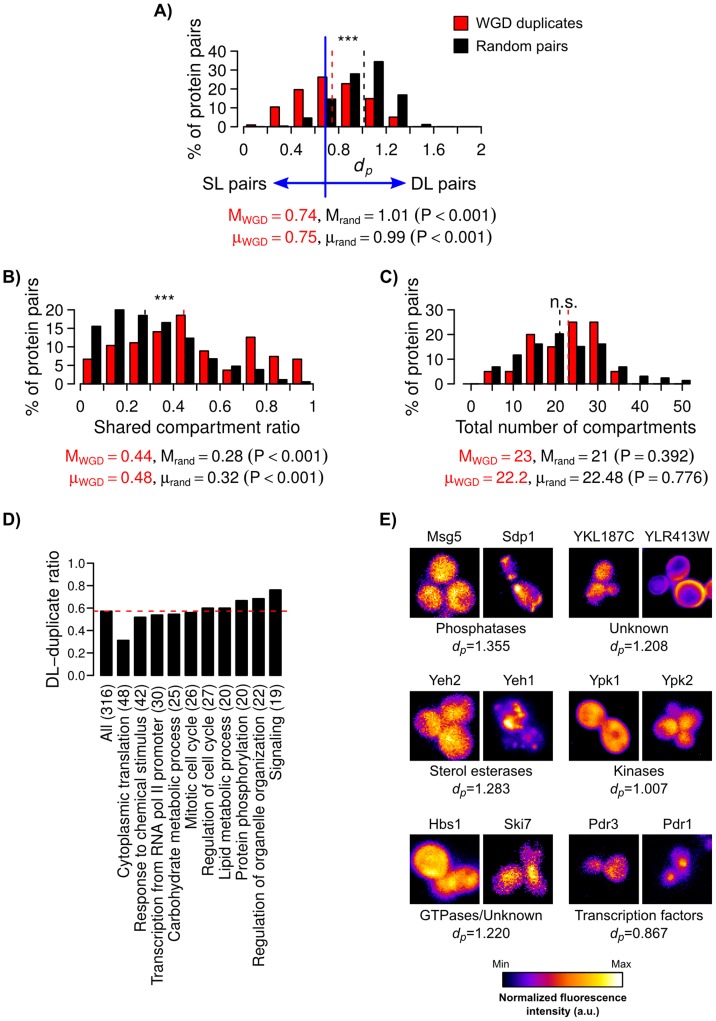
Large portion of WGD duplicates now have diverged localization patterns. Distributions of (**A**) P-profile dissimilarity scores (*d_p_*), (**B**) ratios of shared compartments, and (**C**) numbers of compartments assigned to WGD duplicate (red) and random non-duplicate (black) pairs (M = medians, μ = means of the distributions; two-sided permutation tests for differences in medians or means.) Protein pairs with *d_p_*≥10th-percentile of non-duplicate pairs are referred to as “dissimilarly localized” (DL) pairs, or otherwise as “similarly localized” (SL) pairs. (**D**) Ratios of DL duplicate pairs in the ten biological processes with the highest numbers of duplicate pairs (parentheses = numbers of duplicate pairs with P-profiles, red dashed line = DL-duplicate ratio for all duplicate pairs.) (**E**) Example images from the UCSF dataset [1] showing DL duplicate pairs with different *d_p_* values. The intensity of each image has been scaled to the same range. The molecular functions of the duplicates are also shown if they are known.

To quantify the spatial divergence of WGD duplicates, we determined the *d_p_* for all 326 duplicate pairs that have P-profiles (**Supplementary Dataset S3**), and also the empirical distribution of *d_p_* for 10,000 randomly selected non-duplicate pairs from the proteome (**Fig. 6A**). As expected, we found that WGD duplicates as a whole have significantly lower *d_p_* values and higher shared compartment ratios than random non-duplicates (both P<0.001, two-sided permutation test for difference in medians and means; **Fig. 6A and B**). However, we did not find significant difference in the total numbers of occupied compartments (**Fig. 6C**). We refer to protein pairs with *d_p_* less than a certain percentile of the *d_p_* distribution for random pairs as “similarly localized” (SL) pairs, otherwise as “dissimilarly localized” (DL) pairs. We estimated that ∼42–84% of duplicates are now DL pairs (based on 1st- and 20th-percentile thresholds), which are higher than the ∼24–37% estimated based on UCSF annotations [22]. In the following analyses, we will use a 10th-percent threshold to separate SL and DL pairs. Among the ten biological processes with the highest numbers of duplicates, “cytoplasmic translation” and “signaling” have the lowest and highest DL-duplicate ratios, respectively (**Fig. 6D**). This is consistent with the slower and faster amino acid divergence rates of translational proteins and kinases, respectively, in *S. cerevisiae* [6]. Some of our automatically identified DL duplicates, such as the sterol esterases Yeh1 and Yeh2, protein kinases Ypk1 and Ypk2, and transcription factors Pdr1 and Pdr3, are known to have diverged localizations [40]–[42] (**Fig. 6E**). Thus, our results suggest that a large portion of duplicate proteins have diverged localization patterns.

### Protein relocalization is significantly associated with divergence of biological process but not molecular function

**Figure 7:**
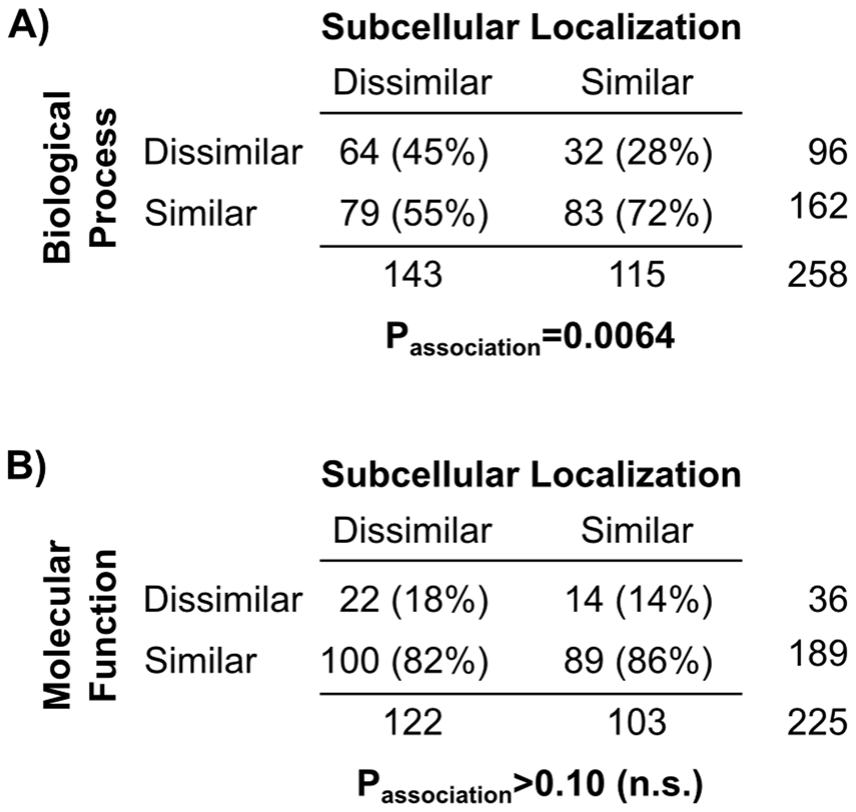
Divergence of protein subcellular localization is significantly associated with divergence of biological process but not molecular function. Contingency tables showing the numbers of SL and DL-pairs with similar or dissimilar (**A**) biological processes or (**B**) molecular functions based on the SGD GOSlim annotations. Only duplicate pairs with both P-profiles and GOSlim annotations were considered. (Duplicate pairs with 75% or more matching functional annotations = “similar” BP or MF pairs, otherwise = “dissimilar” BP or MF pairs; P-values shown were obtained using two-sided Fisher's exact tests.)

Next, we studied if localization and functional divergences are statistically associated events. We considered two different types of functional annotations, namely GO molecular functions and biological processes [8]. Interestingly, we found that protein relocalization in WGD duplicate genes is significantly associated with divergence of biological process (P = 0.0064) but not molecular function (P>0.10, both two-sided Fisher's exact tests; **Fig. 7**). Few DL and SL duplicates have dissimilar molecular functions (18% and 14% respectively, and also see examples in **Fig. 6E**), but ∼45% of DL duplicates are now involved in dissimilar biological processes (**Fig. 7**). To the best of our knowledge, this is the first time that such localization-function relationships are demonstrated systematically and quantitatively at the proteome level. Relocalized duplicates maintain similar molecular functions, likely due to their highly conserved sequences; but they also start to involve in different biological processes, likely due to their interactions with different proteins specific to their occupied compartments. Therefore, our results support the hypothesis that protein relocalization may facilitate functional divergence.

### Spatial divergence is not significantly correlated to number of occupied compartments

**Figure 8:**
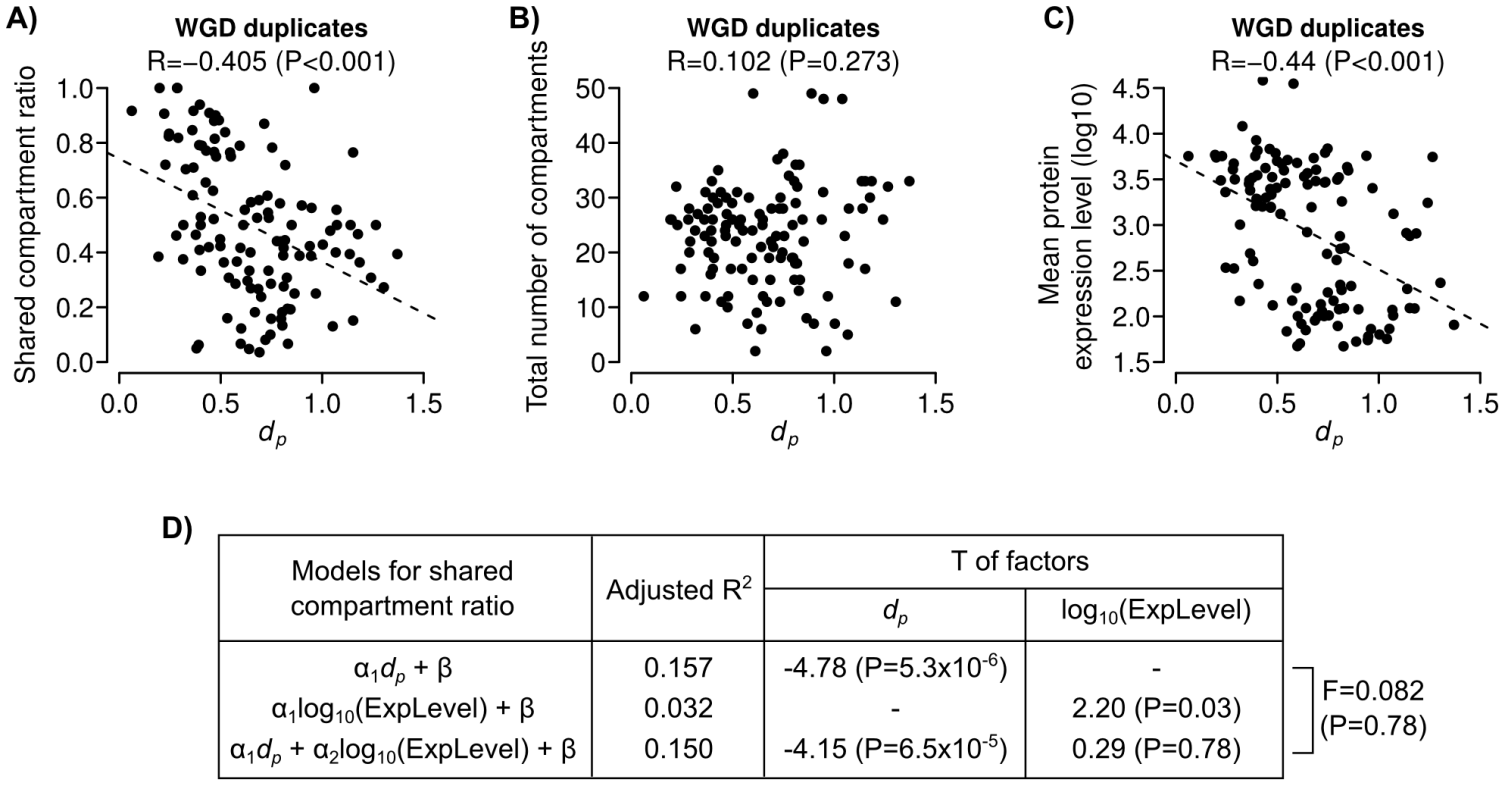
Spatial divergence level of WGD duplicates is significantly correlated to shared compartment ratio but not total number of occupied compartments. Scatter plots showing (**A**) shared compartment ratio, (**B**) total number of compartments, and (**C**) mean protein expression level of WGD duplicates with different *d_p_* values (R = Pearson's correlation coefficient, dashed lines = best linear regression fits of the data.) (**D**) Results from linear regression modeling of shared compartment ratio using *d_p_* and protein expression level as factors (T = T-statistics of the factors; F = F-statistic for the analysis of variance (ANOVA) between two regression models with different factors; R^2^ = squared correlation coefficients between the actual and predicted values of shared compartment ratio.)

Finally, we studied neo- and sublocalization using WGD duplicates. Surprisingly, unlike previous analysis based on UCSF annotations [22], we found that *d_p_* is significantly correlated to shared compartment ratio, but not total number of occupied compartments (P<0.001 and  = 0.286, respectively; **Fig. 8A and B**). We also found that *d_p_* is negatively correlated to protein expression level (P<0.001, **Fig. 8C**), which is consistent with the slow evolutionary rates of highly expressed proteins [43]. However, using regression modeling, we found that protein expression level is not a significant confounding factor between the association of *d_p_* and shared compartment ratio (**Fig. 8D**). Importantly, these results agree with the results that we obtained from duplicates with different divergence times. All of our results could be explained by multi-compartment localizations of ancestral genes. After duplication, different duplicate copies of a gene evolved to develop more specific localization patterns over ancestral compartments. Therefore the total numbers of occupied compartments remain largely unchanged, while the shared compartment ratios are decreasing. Our results suggest that localization divergence was more frequently due to sublocalization.

## Discussion

Using PLAST, we demonstrate that protein subcellular localization can be quantitatively compared and analyzed to reveal protein localization-function relationships that are not obvious under qualitative representations of protein localization. First, protein localization in multiple subcellular compartments is common. Second, spatially-associated proteins tend to participate in related biological processes, and we have identified a repertoire of potentially novel components of biological processes performed at major subcellular compartments. Third, ∼42–84% of duplicate proteins now have diverged localization patterns. Fourth, most of these proteins still perform the same molecular functions, but close to half of them have started to involve in different biological processes, likely due to adaptations to more specific or new subcellular compartments. Fifth, duplicate proteins occupy similar numbers of compartments irrespective of their divergence times or spatial divergence levels, and therefore protein relocalization might be more frequently due to sublocalization. The sublocalization model is very similar to an extensively studied model of functional evolution called “subfunctionalization”, where duplicate genes evolved to complement each other by jointly retaining the full functions of their ancestral genes [44]. However, the sublocalization model does not preclude further functional evolutions via subfunctionalization or other evolutionary mechanisms [45]. Together, our results underscore the importance and utility of quantitative localization profiles in studying protein localization and function.

How can an ancestral gene, or a gene in general, produce proteins localized in multiple compartments? Several molecular mechanisms are possible. First, the budding yeast *Lachancea kluyveri*, which diverged from *S. cerevisiae* before WGD, has a single ortholog to the *S. cerevisiae* SKI7 and HBS1 genes (**Fig. 6E**). This pre-WGD ancestral gene is alternatively spliced to generate two different conserved proteins that can perform similar functions as *S. cerevisiae* Ski7 or Hbs1, respectively [46]. Second, the filamentous fungus *Aspergillus nidulans* has a single NADP-dependent isocitrate dehydrogenase (*idpA*) that has two *Homo sapiens* orthologs (IDH1, IDH2) and three *S. cerevisiae* orthologs (IDP1, IDP2, and IDP3) [47]. Interestingly, this gene has two alternative transcription start sites that specify mitochondrial or cytoplasmic and peroxisomal localizations; whereas the *H. sapiens* IDH1 and IDH2 are specifically localized to cytoplasm and peroxisome or mitochondrion, respectively; and the *S. cerevisiae* IDP1, IDP2, and IDP3 are specifically localized each of these three compartments. Third, several core glycolytic enzymes were also recently found to be targeted to both cytoplasm and peroxisome via alternative splicing and stop codon read-through of the same genes in fungi [48]. Similar transcriptional and/or other post-translational mechanisms [14] may also produce proteins with multi-compartment localization patterns. Quantitative localization profiles are therefore essential to analyze the complex localization patterns of these proteins.

PLAST has several limitations. First, it only works for proteins that can be fluorescently labeled without affecting their localizations. Second, it does not work well for proteins with very low abundance levels. Third, it has poor detection of yeast two-hybrid interactions, likely due to the more transient nature of these interactions [30]. Nevertheless, it can still achieve better overall performances than current protein localization annotations, especially for protein complexes that localized at multiple subcellular compartments. Recent advances in the developments of brighter fluorescent probes and higher-resolution imaging techniques [49] may further improve the performance of PLAST.

As more genome-wide protein localization image datasets become available for different organisms or species [3], [4], PLAST can be readily used to compare protein localizations across different levels of a phylogenetic tree. Protein localization maps can be constructed for cells from different organisms or species using a common set of conserved subcellular compartments. Other possible extensions of PLAST include predicting targeting signals or protein-protein interactions based on proteins that have similar subcellular localization patterns. We have implemented the cell segmentation and feature extraction steps of PLAST using a user-friendly and publicly-available bioimage analysis software platform called “cellXpress” [50], and the P-profile construction and compartment mapping steps using standard R scripts. We have also developed a public web interface (http://plast.bii.a-star.edu.sg) that allows researchers to query protein localization maps created using PLAST. These resources enable systematic and quantitative analyses of protein localization-function relationships, and will help researchers to elucidate protein functions and the causes of their changes.

## Materials and Methods

### Datasets

We used the *S. cerevisiae* GFP image dataset and annotations generated by UCSF [1], the phylogeny of orthologous gene groups estimated by Wapinski et al. [37], the WGD duplicate gene list predicted by Gordon et al. [39], and the AP-MS and Y2H datasets generated by the Dana-Farber Cancer Institute [30]. We retrieved the lists of valid ORFs, GO annotations, GO Slim annotations, GO protein complexes from the Saccharomyces Genome Database (SGD) [28]. Further information about these datasets is included in the **Supplementary Text S1**.

### Overview of our image processing pipeline

For the UCSF yeast GFP dataset, we have developed an image-processing pipeline to estimate cell boundaries based on DIC illumination and DAPI staining. Our method consists of four major steps: background subtraction, image alignment, cell segmentation, and cell combination (**Supplementary Fig. S9**).

### Background subtraction

We minimized non-uniform background intensities in microscopy images using background subtraction. For fluorescence images, we used the rolling ball background subtraction algorithm implemented in ImageJ [51]. We set the rolling ball size to 50 pixels, which is larger than the average diameter of a budding yeast cell in the images. For DIC images, we used ImageJ to convolve the original images with a Gaussian function (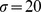) and divide the original images with the convolved images.

### Image alignment

We noticed non-zero lateral offsets between the DIC and fluorescence images (both GFP and DAPI) in the UCSF yeast GFP dataset. These offsets might be due to misalignment of image acquisition instruments. To reduce these artifacts, we have developed a three-step image alignment procedure based on cross-correlation (**Supplementary Fig. S10**). In the first step, a DIC image was segmented to obtain a binary cell mask (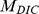) using the Otsu's thresholding method [52], followed by six successive dilations and eight successive erosions with a 3×3 diamond-shape structuring element. In the second step, we estimated the displacement 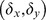 between the binary cell mask and a GFP image using the standard cross-correlation algorithm:
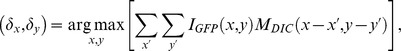
where 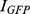 is the fluorescence intensities of the GFP image at position 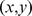. In the third step, we translated the DIC image by 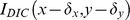 to align it with the GFP image. The same alignment algorithm was also used to align a DAPI image.

### Cell segmentation

After image alignment, we identified individual budding yeast cells from the images (**Supplementary Fig. S9**). First, to suppress high-frequency noise in the images, we smoothed the DIC and DAPI images by convolving them with a 9×9 2D Gaussian lowpass filter (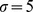). Then, we identified the nuclear regions from the DAPI images using a local structure identification algorithm (see **Local structure identification** section below). We set the algorithm's window size (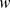) to be 35 pixels, and bias constant (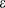) to be −60. Last, to identify cellular regions from the DIC images, we applied the watershed segmentation algorithm implemented in OpenCV to the gradient of the DIC images. The identified nuclear regions were used as initial seeds for the algorithm.

### Local structure identification

We estimated binary local structures using an adaptive thresholding algorithm [53]. Let a 8-bit image of a single budding yeast cell be 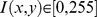 where 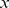 and 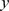 are the x and y coordinates, respectively. We computed an adaptive threshold 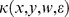 for each pixel in the image using
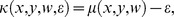
where 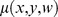 is the mean of the intensity values of a 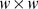 window centered at 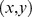, and 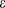 is a user-defined bias constant. Then, we estimated binary local structures, 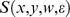, by applying the thresholds to all pixels in the image:
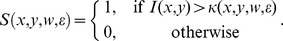



### Cell combination

To reduce oversegmentation, we combined two attached cellular regions if they satisfy the following two criteria:
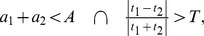
where 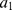 and 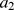 are the areas of the two cellular regions, 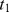 and 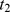 are the total DNA intensity of the two cellular regions, 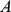 is a threshold for maximum cell area, and 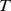 is a threshold for minimum DNA intensity difference between the two regions. For the UCSF yeast GFP dataset, we used 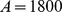 and 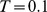.

### Evaluation of cell segmentation

We compared cell masks obtained from our segmentation algorithm, a previous graphical-model-based segmentation algorithm [11], and manual segmentation (**Supplementary Fig. S1**). We selected 20 images with sparsely distributed yeast cells and 20 images with densely distributed yeast cells. These two image sets represent easy and difficult conditions for automated cell segmentation. We expect the segmentation errors of the dense images to be lower than the sparse images. To obtain a ground truth for comparing segmentation errors, we manually segmented each images using a pen-based digitizer (Toshiba M200 laptop). The cell masks for the graphical-model-based algorithm were obtained using the Matlab source code downloaded from http://murphylab.web.cmu.edu/software/2007_Bioinformatics_Yeast/ without any modification.

We used two different segmentation performance criteria: the boundary and Rand error indices [54]. The boundary error index (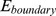) measures the averaged distance between the boundaries of cell masks obtained from manual and automated segmentation, respectively. Smaller boundary error index values mean higher automated segmentation accuracy. We define the boundary error index between two sets of boundary pixels (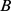 and 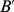) from a manual segmentation mask (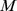) and an automated segmentation mask (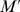), respectively, to be:
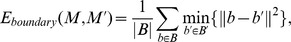
where 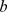 and 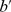 are individual pixels within sets 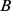 and 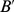, respectively; 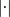 is the cardinality operator; and 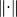 is the Euclidean norm.

We also used the Rand error index [54], which measures the frequency in which the two segmentation masks disagree over whether a pair of pixels belongs to same or different segmented cellular regions. Let us denote the set of labeled regions in a manual segmentation mask 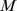 to be 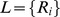 and the set of labelled regions in an automated segmentation mask 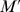 to be 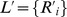, where 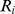 and 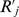 are the *i*-th and *j*-th connected pixels within the respective masks. Furthermore, we denote 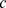 to be the number of pixel pairs in the original image that belongs to the same sets in 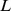 and the same sets in 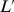, and 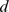 to be the number of pixel pairs in the original image that belongs to different sets in 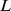 and different sets in 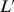. Then, the Rand error index is:
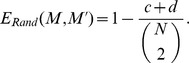
where *N* is the total number of pixels in the original image.

### Identification of subcellular regions

Based on the cellular and nuclear (DNA) regions identified from our segmentation algorithm (see **Cell Segmentation** above), we defined three additional subcellular regions, namely cytoplasmic (non-DNA), cytoplasmic boundary, and inner cytoplasmic regions (**Supplementary Fig. S11**). The cytoplasmic region was computed by subtracting the nuclear regions from the cellular regions. The cytoplasmic boundary region was computed from the cytoplasmic region using a binary erosion operator with a circular structuring element (radius = 3 pixels). Finally, the inner cytoplasmic region was computed by subtracting the cytoplasmic boundary region from the cytoplasmic region.

### Single-cell intensity normalization

To eliminate the effects of protein-to-protein variations in expression levels and only consider protein spatial localization patterns, we divided the GFP intensity values of all the pixels within the detected cellular regions (see **Identification of subcellular regions** above) of individual yeast cells to their sums. So, the total GFP intensity of each normalized cell became one. All features were extracted only after this normalization step.

### Feature extraction

We extracted 623 features from the normalized GFP images of each budding yeast cell. They include 81 morphology, 45 intensity, 20 intensity ratio, 273 Haralick texture [55], 18 moment [56], and 186 local structure features of the five identified subcellular regions. Most of these features were commonly used to describe protein subcellular localization patterns [57]. Local structure features are developed by us for PLAST, and described in more detail below.

### Local structure features

We have designed a new feature type, called “local structures”, to describe protein distribution patterns in local subcellular regions. Extraction of local structure features consists of three steps: local structure identification, global ratio feature computation, and stepwise ratio feature computation. In the first step, we applied the local structure detection algorithm (see **Local structure identification** above) to identify local structures from the GFP images. A smaller window size (*w*) will extract finer local structures, while a larger window size will extract coarser global structures in the protein localization patterns. For the UCSF yeast GFP dataset, we set 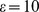 and found that the local structures of most yeast cells converged to similar global patterns after *w* = ∼33 (**Supplementary Fig. S12**). Therefore we only used 16 different window sizes (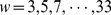 pixels) for feature extraction.

In the second step, we extracted six features (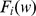 where 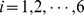) based on the identified local structures of window size 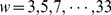. These features are total GFP intensity, mean GFP intensity, standard-deviation GFP intensity, binary object number, binary object total area, and binary object mean area. We also extracted the same features (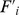) but based on the cellular regions of the same images. Then, we computed global ratio features (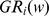) of the local structures using
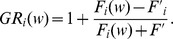
These features are designed to detect the concentrations of GFP signals in the identified local structures. For the UCSF yeast GFP dataset, this amounted to 6 features per window size×16 window sizes per cell = 96 features per cell.

In the third step, we computed the stepwise ratios (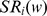) of the same six features between local structures of two consecutive window sizes, namely
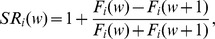
where 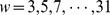. These ratios are designed to detect changes in the concentrations of GFP signals from finer to coarser local structures (**Supplementary Fig. S12**). For the UCSF yeast GFP dataset, this amounted to 90 additional features per cell. Therefore, the total number of local structure features was 96+90 = 186.

### Quality control

To automatically remove badly or wrongly segmented cells, we used two quality control criteria, namely cell area and solidity (cell area/convex hull area). First, we removed segmented cells that have cell area <300 pixels or >2000 pixels. Then, we removed segmented cells that have solidity value >0.2. There were 33 strains with more than one image set, and for each strain we used only one image set. We removed three yeast strains (YDL125C, YJL026W, YLR109W) that have abnormal image size (58×58 pixels). After feature extraction, there were 15 cells with NaN values, and we replaced these values with the medians across all cells in each of the yeast strains. We obtained ∼20 segmented cells per yeast strain on average. Only one of the yeast strains, namely YHR011W, has less than two cells, and thus the whole strain was removed from further analysis. We also removed 55 strains that have mislocalized proteins due to the GFP tags [1], and 34 strains that have invalid ORFs according to SGD (see **Datasets**). The final number of yeast strains that we used in our study was 4066.

### P-profile_SVM_ construction

To construct P-profiles_SVM_ for yeast strains that have been labeled for a protein, we used a SVM [16] with a linear kernel function that has good performance in many real classification problems [58], [59]. The decision function of the SVM is given by
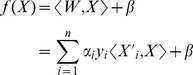
where 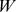 is a normal vector to the decision hyperplane (**Supplementary Fig. S2A**), 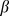 is a bias term, 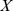 is an input sample, 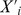 is a training sample, 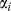 is a coefficient determined by the SVM algorithm, *y_i_* is the class label of the *i*-th training sample, 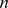 is the number of training sample, and 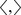 is the dot product operator. We trained the SVM to find the optimum *W* that can separate yeast cells labeled for a protein, and a fixed set of reference cells. Then, we divided *W* with the sum of all its elements so that it becomes a unit vector. A SVM algorithm with linear kernel function has two main parameters, the cost parameter for constraint violation (*cost*), and the tolerance of termination criterion (*epsilon*). We set epsilon to its default value, 0.01. For each yeast strain, a grid search on the values of 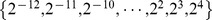 was performed to determine the cost parameter with the maximum training classification accuracy between the two set of cells. We used the SVM training algorithm implemented in the “LiblinearR” v1.80-6 package [60] under the R environment.

To generate the reference cell set, we first randomly sampled 9 sets of reference cells (with 10, 20, 30, …, or 90 cells) from yeast strains that have been assigned to four of the largest UCSF categories, namely “cytoplasm”, “nucleus”, “mitochondrion”, and “endoplasmic reticulum”. Then, based on each of the reference sets, we constructed a set of P-profiles (SVM) for all the yeast strains, and trained a multi-class SVM classifier (see **Supervised classification of UCSF categories**) to classify 2654 ORFs with single UCSF category assignments. The evaluation process was repeated five times with different random selections of reference cell sets, and we chose the set of P-profiles with the highest average accuracy in classifying the 2654 ORFs to represent protein subcellular localization (**Supplementary Fig. S2B**).

### Supervised classification of UCSF categories

We used a multi-class SVM with linear kernel proposed by Crammer and Singer [61] and implemented in the “LiblinearR” v1.80-6 package under the R environment. Similar to two previous supervised learning studies of the UCSF yeast GFP datasets [11], [13], we used six fold cross validation with six random trials to estimate the classification accuracy for all UCSF categories. In order to make our results comparable to these two previous studies, we performed the supervised classification analysis only on the 2654 yeast strains that had been assigned to single subcellular compartments by UCSF, and without the quality control step as described in the **Quality Control** section.

### P-profile dissimilarity score

We computed the dissimilarity score between two P-profiles *h* and *g* as
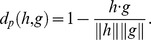
To compare the P-profiles of a protein (*h*) and a group of proteins (*G*), we took the mean of all the pairwise dissimilarity scores:
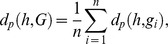
where 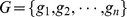.

### Affinity propagation clustering

Affinity propagation is an algorithm that identifies representative data points, called “exemplars”, and forms clusters of data points around these exemplars [29]. It starts by considering all data points as potential exemplars, and exchange messages between data points until convergence of a set of exemplars or clusters. Preferences of data points are used to control the number of selected exemplars. Low or high preference values lead to low or high numbers of clusters, respectively. To cluster P-profiles, we used the same preference values for all data points, and varied the values to determine the optimum number of clusters (**Supplementary Fig. S3A**). We used the affinity propagation clustering algorithm implemented in “apcluster” v1.3.2 package under the R environment.

### Hierarchical clustering of P-profiles

We used the standard agglomerative hierarchical clustering algorithm implemented in the hclust() function under the R environment. The P-profile dissimilarity scores and Ward agglomeration method were used.

### Precision, recall and F1-score

The definitions of precision, recall, and F1-scores are:
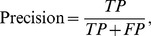


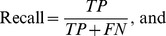


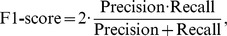
where 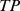 is the number of true positives, 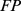 is the number of false positive, and 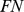 is the number of false negatives. F1-score is the harmonic mean of precision and recall. All of these three criteria are commonly used to measure information retrieval performances [32].

For each profiling/annotation method and each protein complex, we measured the maximum F1-score of the method across all the tested query-protein sizes for the protein complex. To compare the performances of two different methods, we performed a paired t-test between the maximum F-1 scores of the methods obtained from all the protein complexes. The p-values for all pair-wise comparisons of the four methods (P-profile_SVM_, P-profile_mean_, UCSF and SGD Go Slim) were Bonferroni adjusted.

### Subunit search based on UCSF or SGD GOSlim annotations

Given a set of known subunits of a protein complex, we identified the UCSF or SGD GOSlim cellular component category associated with the highest number of subunits. Then, we predicted all other ORFs annotated with this category to be the other “subunits” of the complex, and measured the corresponding precision, recall and F1 scores.

### Catalog of subcellular compartments

To construct a localization map, we used a catalog of subcellular compartments that consists of known protein components of 23 major organelles and 50 large protein complexes. The list of protein components for major organelles is based on the manually curated SGD GoSlim cellular component dataset (see **Datasets**). The original dataset has 25 categories. We removed the “cellular_component”, “microtubule organizing center”, “extracellular region”, “unknown”, and “other” categories, and included the “lipid particle”, “endosome”, and “nuclear envelope” categories. The list of components for large protein complexes is based on the manually curated SGD GO protein complex dataset (see **Datasets**). The original dataset has 416 protein complexes. We only considered protein complexes with at least 15 subunits, and removed the “microtubule organizing center” complex because it overlaps with the “spindle pole body” complex.

### Compartment assignment

For each ORF, we systematically queried the P-profile database for the *d_p_* scores between the ORF and all the compartments (see Catalog of subcellular compartments above). Then, we removed outliers by trimming the upper and lower fifth percentiles of the obtained *d_p_* scores, and estimated the probability distribution of the retained scores using the kernel density estimation method, density(), implemented in the R environment. We identified the local maximum of the distribution with the largest *d_p_* value, and used the *d_p_* value as the mean (*μ_null_*) of the distribution of non-specifically localized compartments. We assumed the non-specific distribution is Gaussian, and estimated its standard deviation (*σ_null_*) from all *d_p_* scores larger than *μ_null_*. Then, we standardized all the *d_p_* scores using 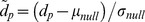, and also calculated their corresponding P-values based on a normalized non-specific distribution with zero mean and unit variance. Finally, we performed Bonferroni correction on all the obtained P-values, and assigned compartments with adjusted P-values less than a given threshold to the ORF.

### Duplicates with different divergence times

We obtained a phylogeny of orthologous gene groups estimated for seventeen Ascomycota fungi [37]. We only used *S. cerevisiae* duplicates that could be traced to their originating ancestors without any loss events. The approximate divergence ages of the phylogenetic tree are based on past estimations [38], [62], [63], and only used for visualization in **Fig. 5**. In our analysis, we always divided the duplicates into two groups: “old” (pre-WGD) or “young” (other) duplicates, and never use the estimated values of these divergence ages.

### Permutation test for difference in means or medians

To perform a non-parametric test for the statistical significance of the observed difference in means or medians between two sets of values, we randomly permutated the labels of the datasets for 10,000 times. For each permutation, we measured the mean or median difference between the two sets according to the permutated labels. The P-value was estimated to be the fraction of permutations with absolute mean or median difference larger than the observed absolute mean or median difference.

### Software implementation and availability

We have developed “cellXpress” v1.10 (http://www.cellXpress.org) to perform all the image processing and feature extraction steps [50]. The software package is general and could also be used to process images of other cell types. The extracted features were then loaded and processed using the R computing environment v3.0.1 under Gentoo Linux operating system. All the R source code for PLAST and datasets that we used in this study can be downloaded from http://plast.bii.a-star.edu.sg.

## Supporting Information

Dataset S1 **Global subcellular localization map of the budding yeast** ***Saccharomyces cerevisiae*** **proteome.**
(CSV)

Dataset S2 **List of proteins assigned with select subcellular compartments.**
(CSV)

Dataset S3 **P-profile dissimilarity scores and numbers of subcellular compartments localized by WGD duplicate genes.**
(CSV)

Figure S1 **A DIC-based segmentation algorithm for budding yeast cells.** Example images from the UCSF dataset [1] showing DIC and GFP channels overlaid with cell boundaries detected using manual segmentation, Chen07's graphical model method [11], and PLAST on (**A**) sparse or (**B**) dense populations of budding yeast cells (white lines = detected cell boundaries). The overall boundary and Rand error indices are shown in the lower panel for these two conditions (n.s. = P>0.05, *** = P<0.001, n = 20 images, two sided t-test).
(TIF)

Figure S2 **Construction of profile vectors from single-cell feature measurements.** (**A**) Schematic showing how P-profiles are constructed to represent cells labeled for a protein. In the m-dimensional feature space, each cell is represented by a vector (red circles). “P-profile_mean_” is the mean or centroid (white arrow in left panel) of all feature vectors for the cells labeled for the protein. “P-profile_SVM_” is a unit vector (white arrow in right panel) orthogonal to a hyperplane that optimally divides the cells labeled for the protein (red circles) and a fixed set of reference cells (blue circles). The hyperplane is determined using a linear support vector machine (SVM). (**B**) Classification accuracies of all the randomly selected sets of reference cells. Please refer to **P-profile_SVM_ construction** for the procedures to generate these reference sets. The maximum and final selected reference cell set is circled in red. (**C**) Comparisons of P-profiles_SVM_, P-profiles_mean_, and quantiative features generated by two previous analysis frameworks (“Chen07” and “Huh09”) [11], [13] in classifying 2654 ORFs with single UCSF category assignments.
(TIF)

Figure S3 **Clustering of P-profiles using an affinity propagation algorithm.** (**A**) Number of clusters (or exemplars) selected by an affinity propagation algorithm as a function of preference value. We chose to divide all the P-profiles into 20 clusters, before the number of clusters started to increase dramatically. Each cluster was named according to its most enriched UCSF category (see **Fig. S4**; NC = “nucleus”, NL = “nucleolus”, CP = “cytoplasmic”, MC = “mitochondrial”, ER = “endoplasmic reticulum”, PM = “plasma membrane”, and AB = “ambiguous”). (**B**) Microscopy images from the UCSF dataset [1] showing the final exemplars selected by the affinity propagation algorithm. The intensity levels of each image have been scaled to the same range to show protein subcellular localization patterns. The ORF, protein name (if known), and the UCSF categories of the exemplars are shown below their cluster names.
(TIF)

Figure S4 **Automated clustering of P-profiles reveals novel localization patterns.** Heatmaps showing P-values for the enrichments of (**A**) UCSF categories or (**B**) selected significantly enriched GO biological process categories (P<0.001) in the 20 identified clusters (**Fig. 1D** **and S3**) as determined by one-sided hypergeometric tests. Each cluster is labeled according to its most enriched UCSF category. The total number of proteins in each UCSF or GO biological process category is listed in parenthesis after the category name.
(TIF)

Figure S5 **Normalized differences between F1-scores are weakly correlated to protein complex sizes.**
(TIF)

Figure S6 **Examples of P-profile dissimilarity score distributions for non-specifically localized compartments.** (Black curves = probability distributions of the *d_p_* values between 10 randomly chosen ORFs and all 73 major subcellular compartments; red dashed lines and curves = estimated means and distributions, respectively, of the *d_p_* values between the ORFs and non-specifically localized compartments; blue lines = Bonferroni-adjusted P-value thresholds of 2.5×10^−4^).
(TIF)

Figure S7 **Subcellular localization map for the** ***Saccharomyces cerevisiae*** **proteome.** A subcellular localization map showing the standardized P-profile dissimilarity scores (*d_p_*) between 4066 ORFs (x-axis) and a comprehensive catalog of 73 major subcellular compartments (y-axis) in a yeast cell. The compartments (rows) were ordered using a hierarchical clustering algorithm with cosine dissimilarity scores, and labeled with color codes according to their known functions or localizations (“common” compartments = compartments assigned to large numbers of ORFs.)
(TIF)

Figure S8 **ORFs assigned with cytosolic ribosome are enriched with ORFs co-purified with cytosolic ribosome.** We obtained the list of ORFs that co-purified with cytosolic ribosome from [36]. Shown are the numbers of ORFs in different subsets of the data, and the P-values obtained from hypergeometric tests.
(TIF)

Figure S9 **An image-processing pipeline to segment budding yeast cells.**
(TIF)

Figure S10 **Automated alignment of DIC and fluorescence images based on cross-correlation.**
(TIF)

Figure S11 **Example images showing the five subcellular regions identified using PLAST.**
(TIF)

Figure S12 **Example of binary local structures detected using different window sizes.**
(TIF)

Text S1 **Sources of the datasets that we used in our study.**
(DOCX)
