# Supplementary figures and images for: Quantitative Protein Localization Signatures Reveal an Association between Spatial and Functional Divergences of Proteins
Source: PLoS Comput Biol. 2014 Mar 6;10(3):e1003504. doi: 10.1371/journal.pcbi.1003504 (PMC3945119; doi:10.1371/journal.pcbi.1003504)

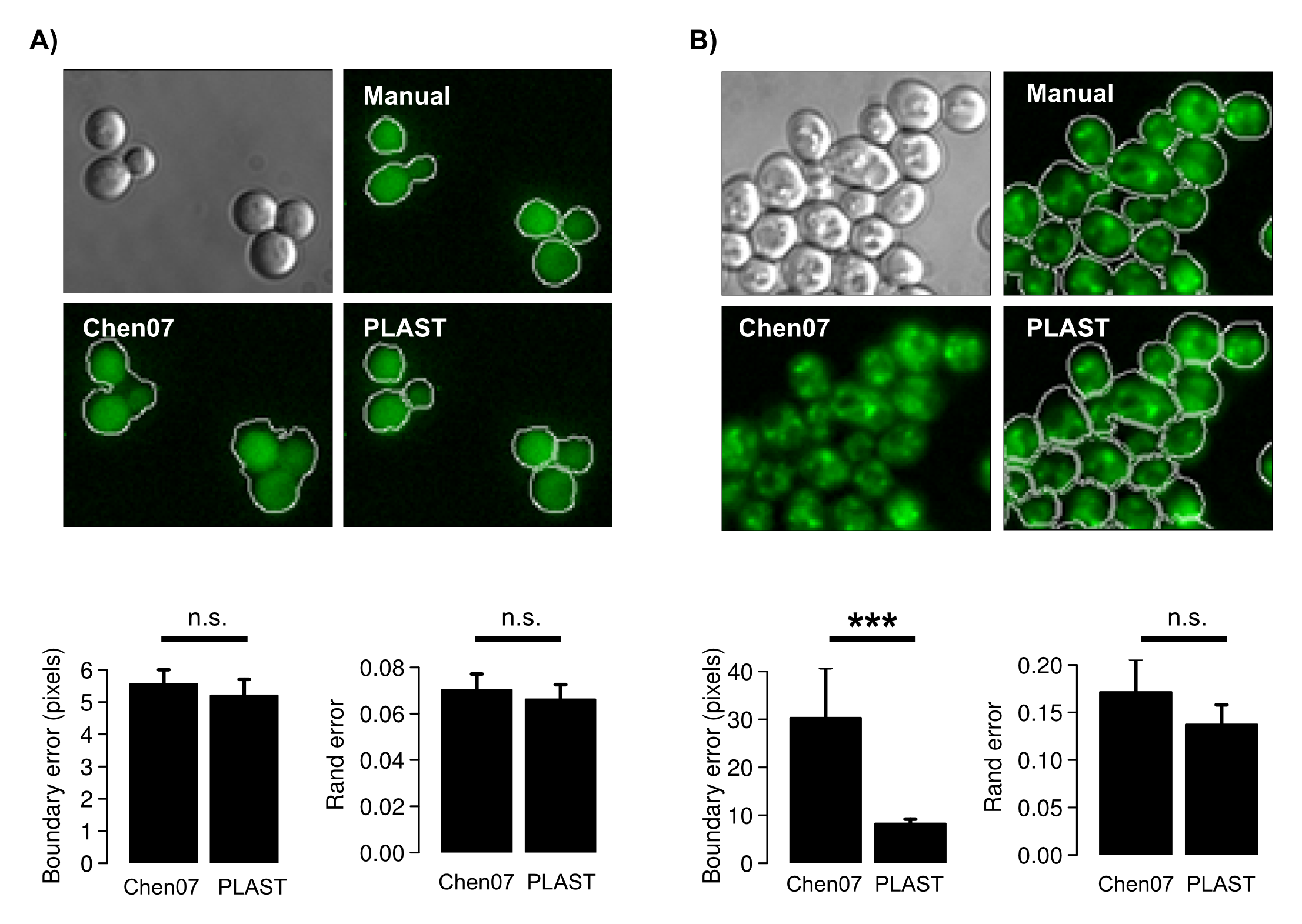

Supplement: Figure S1 — A DIC-based segmentation algorithm for budding yeast cells. Example images from the UCSF dataset [1] showing DIC and GFP channels overlaid with cell boundaries detected using manual segmentation, Chen07's graphical model method [11], and PLAST on (A) sparse or (B) dense populations of budding yeast cells (white lines = detected cell boundaries). The overall boundary and Rand error indices are shown in the lower panel for these two conditions (n.s. = P>0.05, *** = P<0.001, n = 20 images, two sided t-test). (TIF) [file pcbi.1003504.s004.tif]

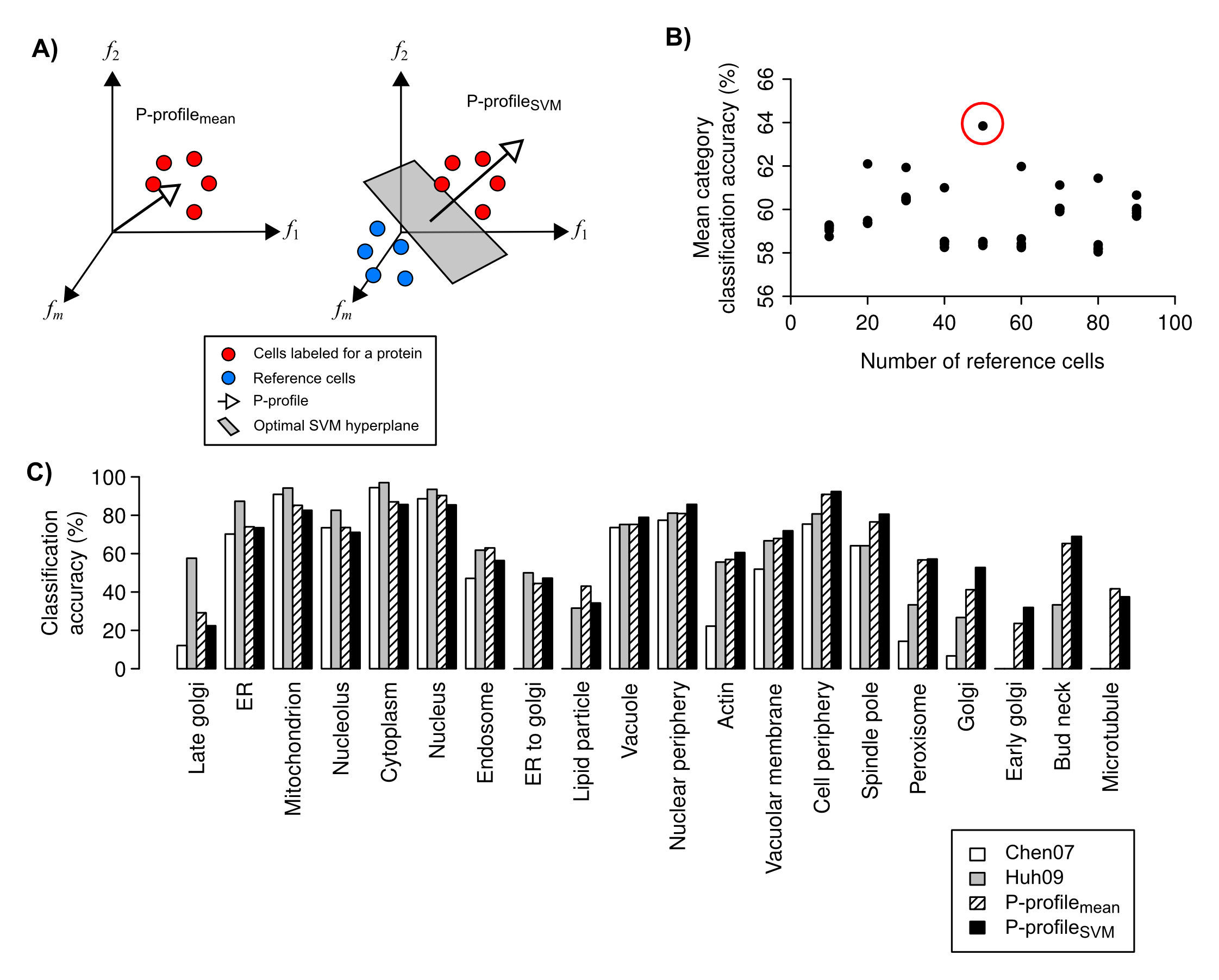

Supplement: Figure S2 — Construction of profile vectors from single-cell feature measurements. (A) Schematic showing how P-profiles are constructed to represent cells labeled for a protein. In the m-dimensional feature space, each cell is represented by a vector (red circles). “P-profilemean” is the mean or centroid (white arrow in left panel) of all feature vectors for the cells labeled for the protein. “P-profileSVM” is a unit vector (white arrow in right panel) orthogonal to a hyperplane that optimally divides the cells labeled for the protein (red circles) and a fixed set of reference cells (blue circles). The hyperplane is determined using a linear support vector machine (SVM). (B) Classification accuracies of all the randomly selected sets of reference cells. Please refer to P-profileSVM construction for the procedures to generate these reference sets. The maximum and final selected reference cell set is circled in red. (C) Comparisons of P-profilesSVM, P-profilesmean, and quantiative features generated by two previous analysis frameworks (“Chen07” and “Huh09”) [11], [13] in classifying 2654 ORFs with single UCSF category assignments. (TIF) [file pcbi.1003504.s005.tif]

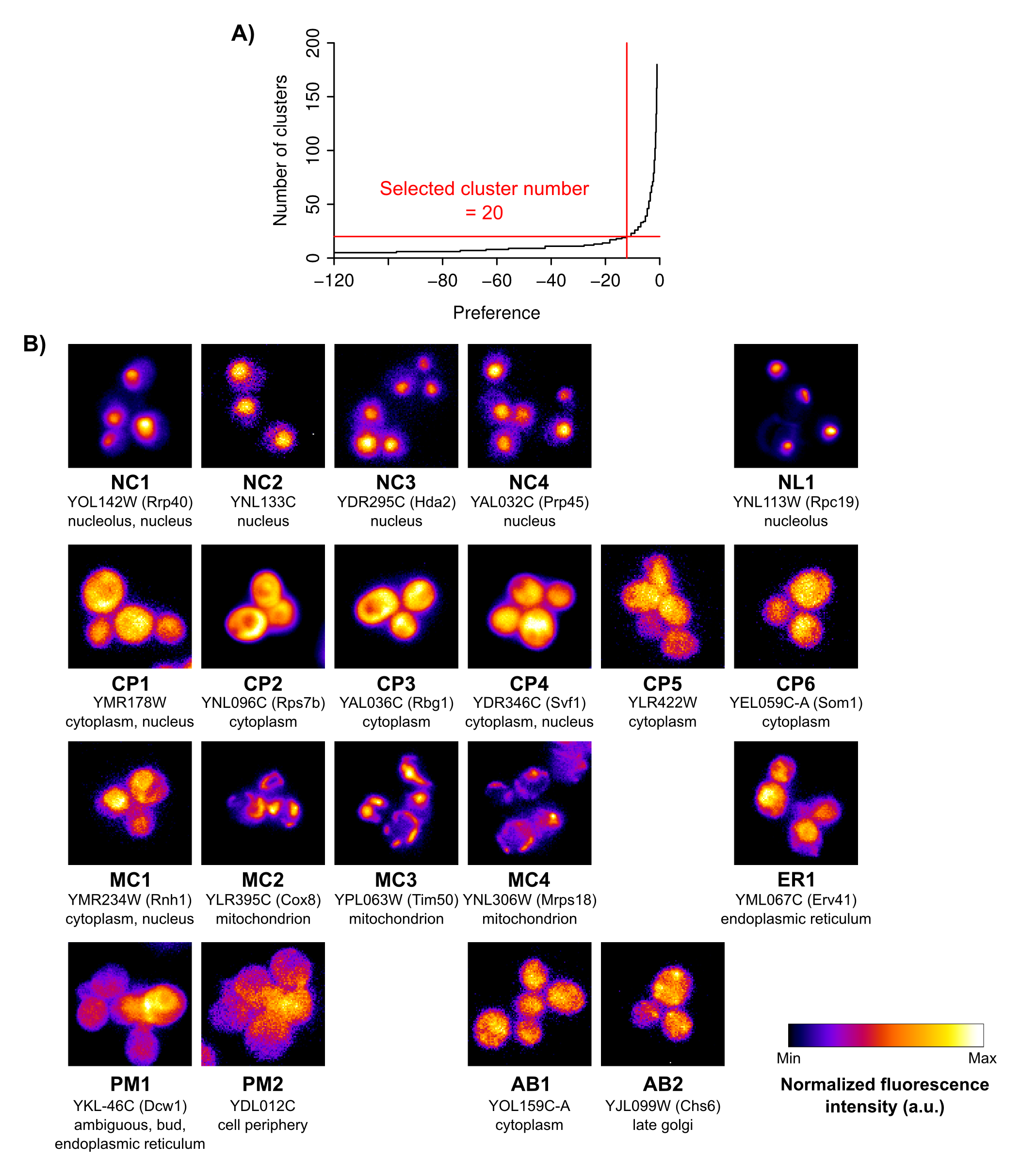

Supplement: Figure S3 — Clustering of P-profiles using an affinity propagation algorithm. (A) Number of clusters (or exemplars) selected by an affinity propagation algorithm as a function of preference value. We chose to divide all the P-profiles into 20 clusters, before the number of clusters started to increase dramatically. Each cluster was named according to its most enriched UCSF category (see Fig. S4; NC = “nucleus”, NL = “nucleolus”, CP = “cytoplasmic”, MC = “mitochondrial”, ER = “endoplasmic reticulum”, PM = “plasma membrane”, and AB = “ambiguous”). (B) Microscopy images from the UCSF dataset [1] showing the final exemplars selected by the affinity propagation algorithm. The intensity levels of each image have been scaled to the same range to show protein subcellular localization patterns. The ORF, protein name (if known), and the UCSF categories of the exemplars are shown below their cluster names. (TIF) [file pcbi.1003504.s006.tif]

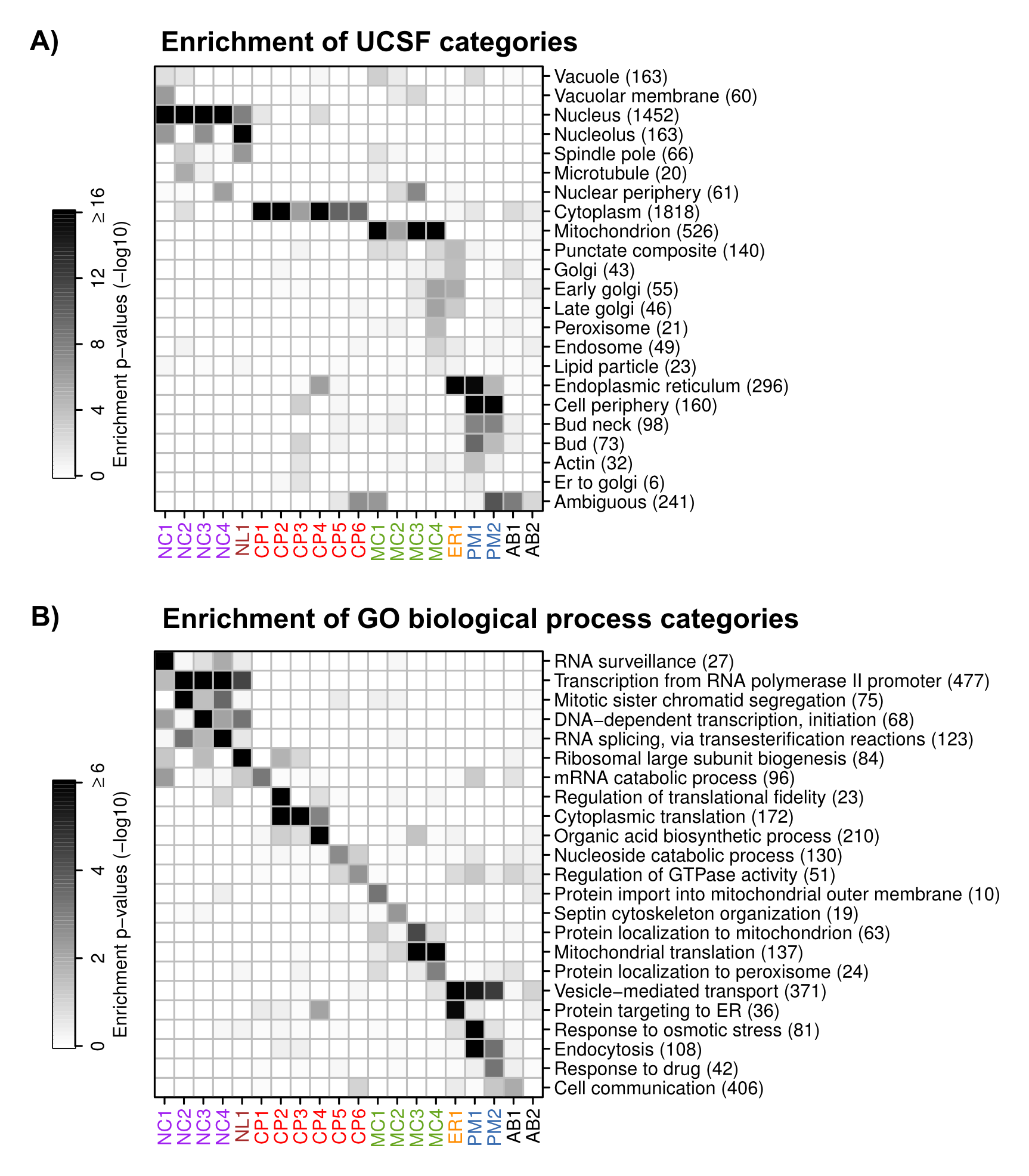

Supplement: Figure S4 — Automated clustering of P-profiles reveals novel localization patterns. Heatmaps showing P-values for the enrichments of (A) UCSF categories or (B) selected significantly enriched GO biological process categories (P<0.001) in the 20 identified clusters ( Fig. 1D and S3) as determined by one-sided hypergeometric tests. Each cluster is labeled according to its most enriched UCSF category. The total number of proteins in each UCSF or GO biological process category is listed in parenthesis after the category name. (TIF) [file pcbi.1003504.s007.tif]

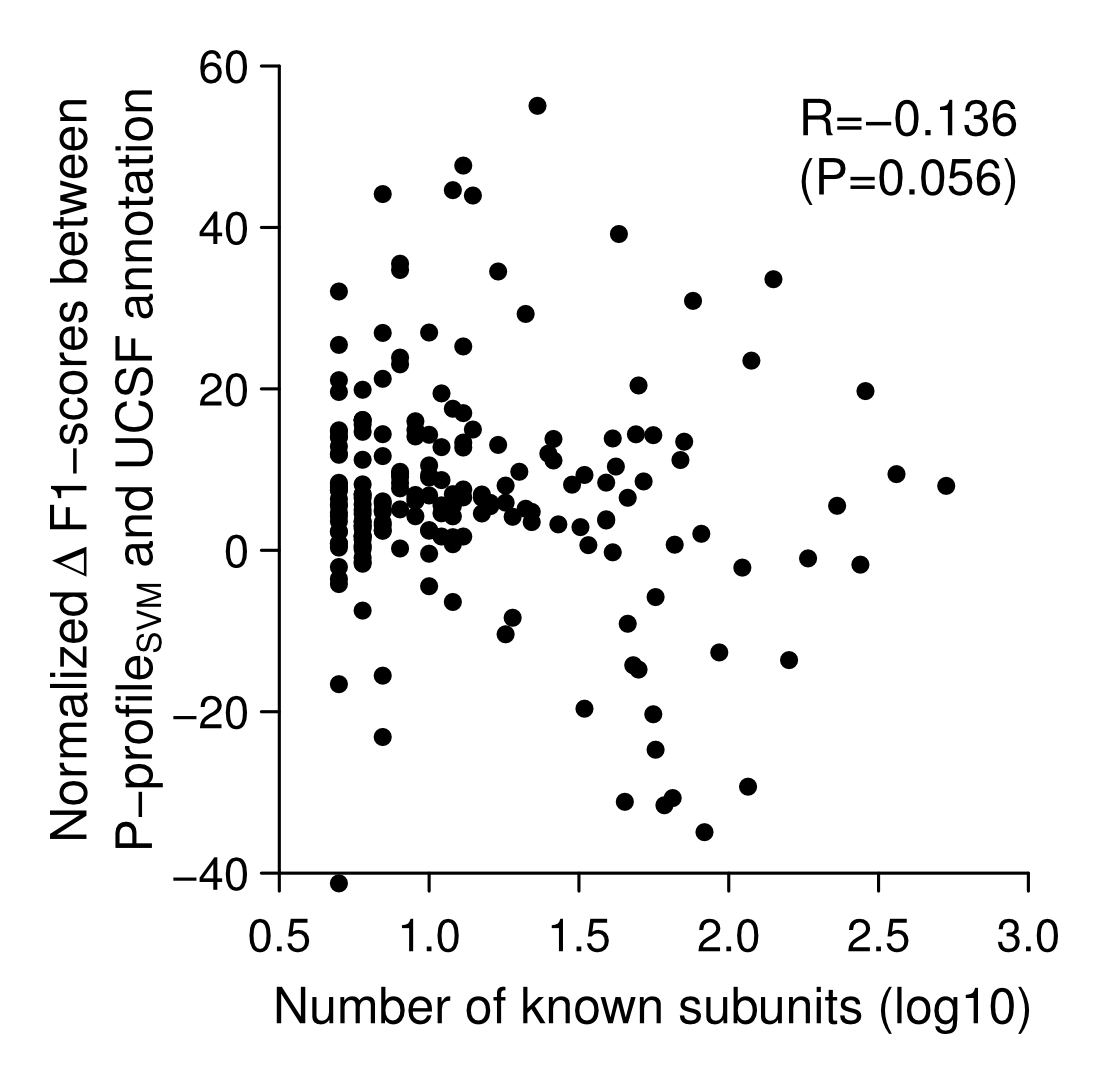

Supplement: Figure S5 — Normalized differences between F1-scores are weakly correlated to protein complex sizes. (TIF) [file pcbi.1003504.s008.tif]

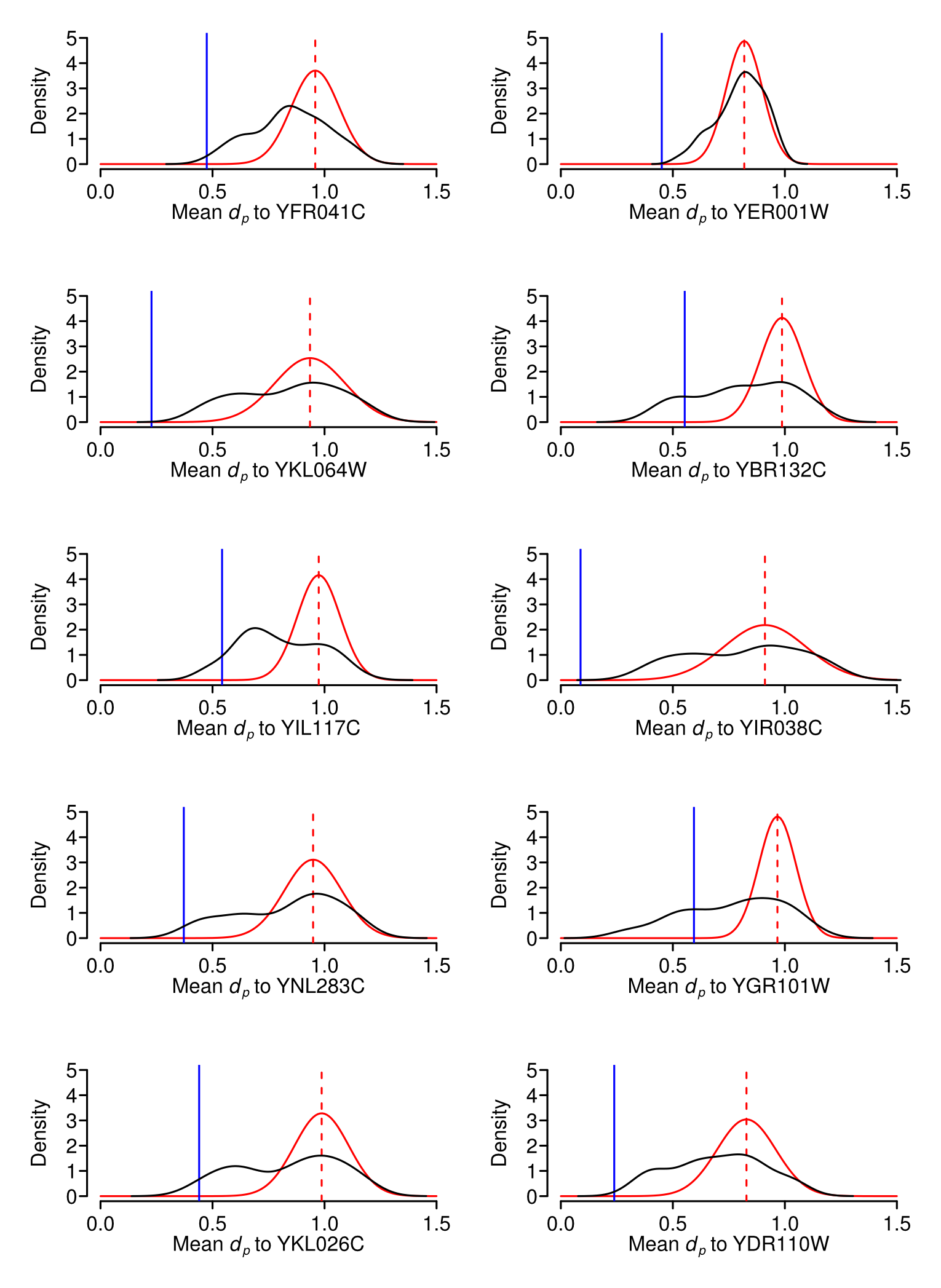

Supplement: Figure S6 — Examples of P-profile dissimilarity score distributions for non-specifically localized compartments. (Black curves = probability distributions of the dp values between 10 randomly chosen ORFs and all 73 major subcellular compartments; red dashed lines and curves = estimated means and distributions, respectively, of the dp values between the ORFs and non-specifically localized compartments; blue lines = Bonferroni-adjusted P-value thresholds of 2.5×10^−4^). (TIF) [file pcbi.1003504.s009.tif]

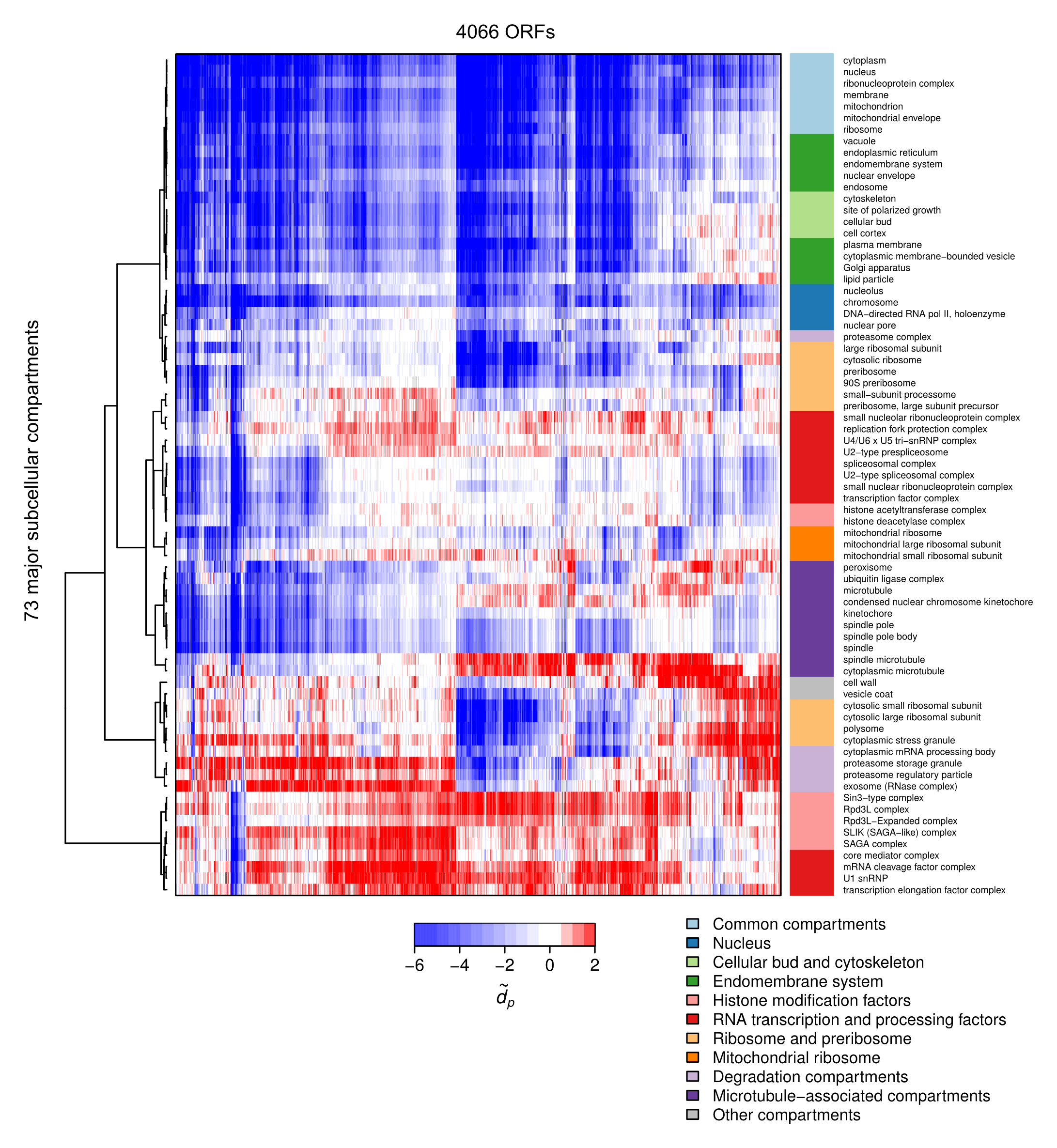

Supplement: Figure S7 — Subcellular localization map for the Saccharomyces cerevisiae proteome. A subcellular localization map showing the standardized P-profile dissimilarity scores (dp) between 4066 ORFs (x-axis) and a comprehensive catalog of 73 major subcellular compartments (y-axis) in a yeast cell. The compartments (rows) were ordered using a hierarchical clustering algorithm with cosine dissimilarity scores, and labeled with color codes according to their known functions or localizations (“common” compartments = compartments assigned to large numbers of ORFs.) (TIF) [file pcbi.1003504.s010.tif]

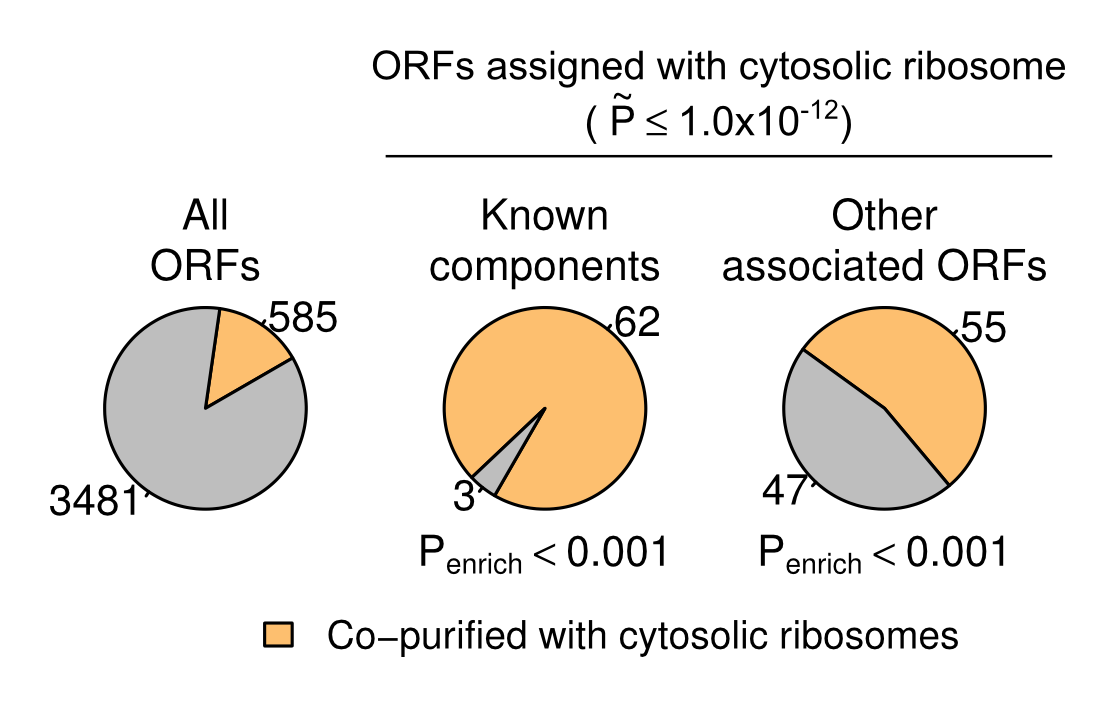

Supplement: Figure S8 — ORFs assigned with cytosolic ribosome are enriched with ORFs co-purified with cytosolic ribosome. We obtained the list of ORFs that co-purified with cytosolic ribosome from [36]. Shown are the numbers of ORFs in different subsets of the data, and the P-values obtained from hypergeometric tests. (TIF) [file pcbi.1003504.s011.tif]

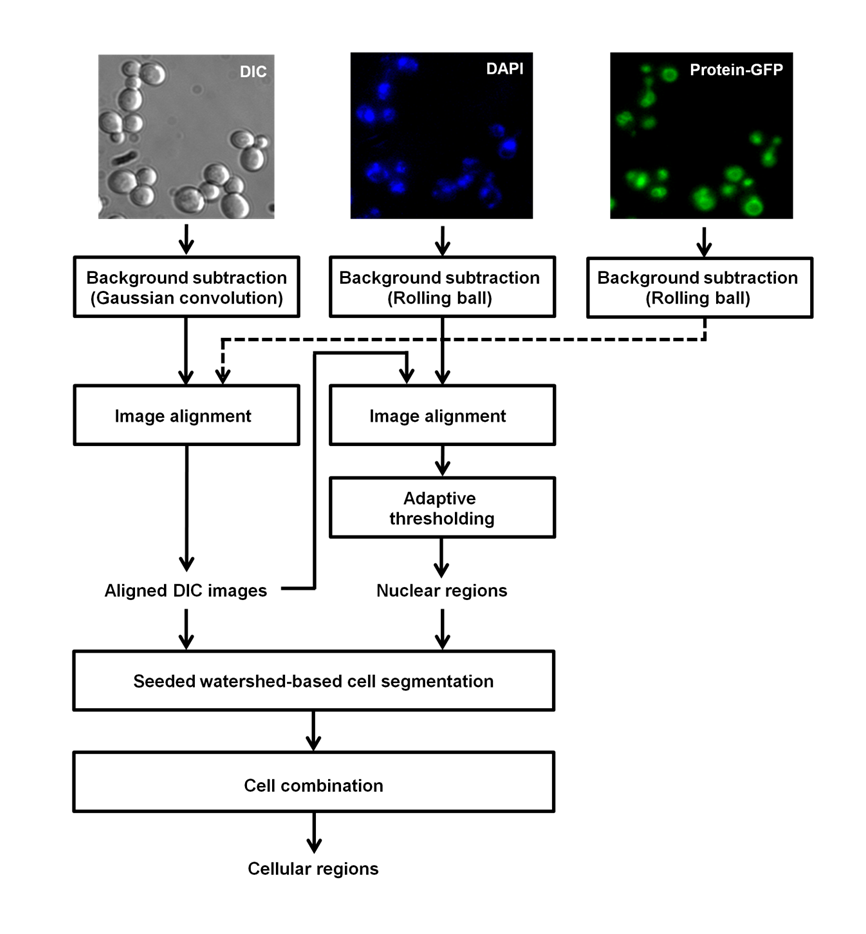

Supplement: Figure S9 — An image-processing pipeline to segment budding yeast cells. (TIF) [file pcbi.1003504.s012.tif]

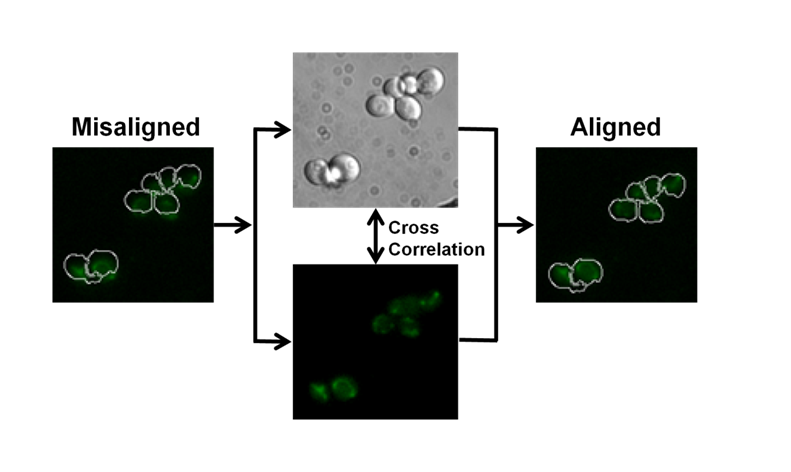

Supplement: Figure S10 — Automated alignment of DIC and fluorescence images based on cross-correlation. (TIF) [file pcbi.1003504.s013.tif]

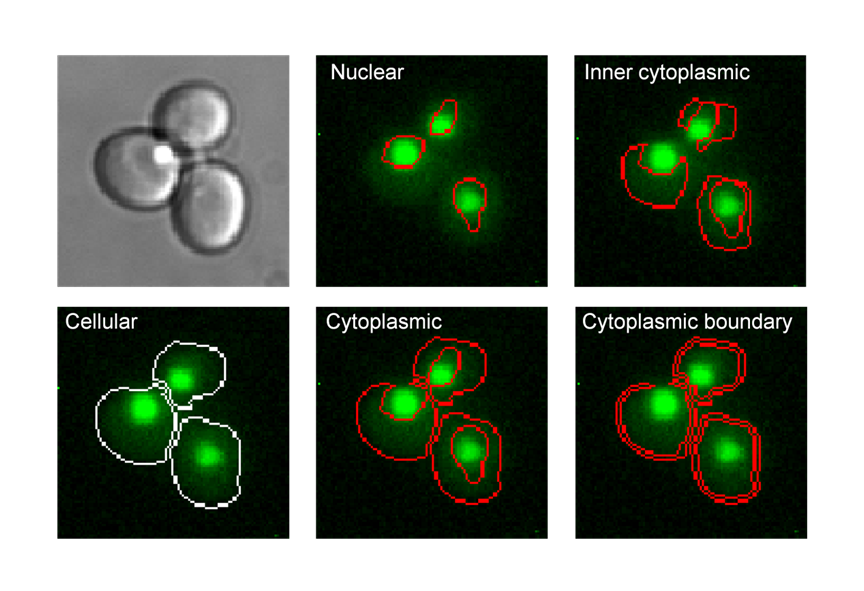

Supplement: Figure S11 — Example images showing the five subcellular regions identified using PLAST. (TIF) [file pcbi.1003504.s014.tif]

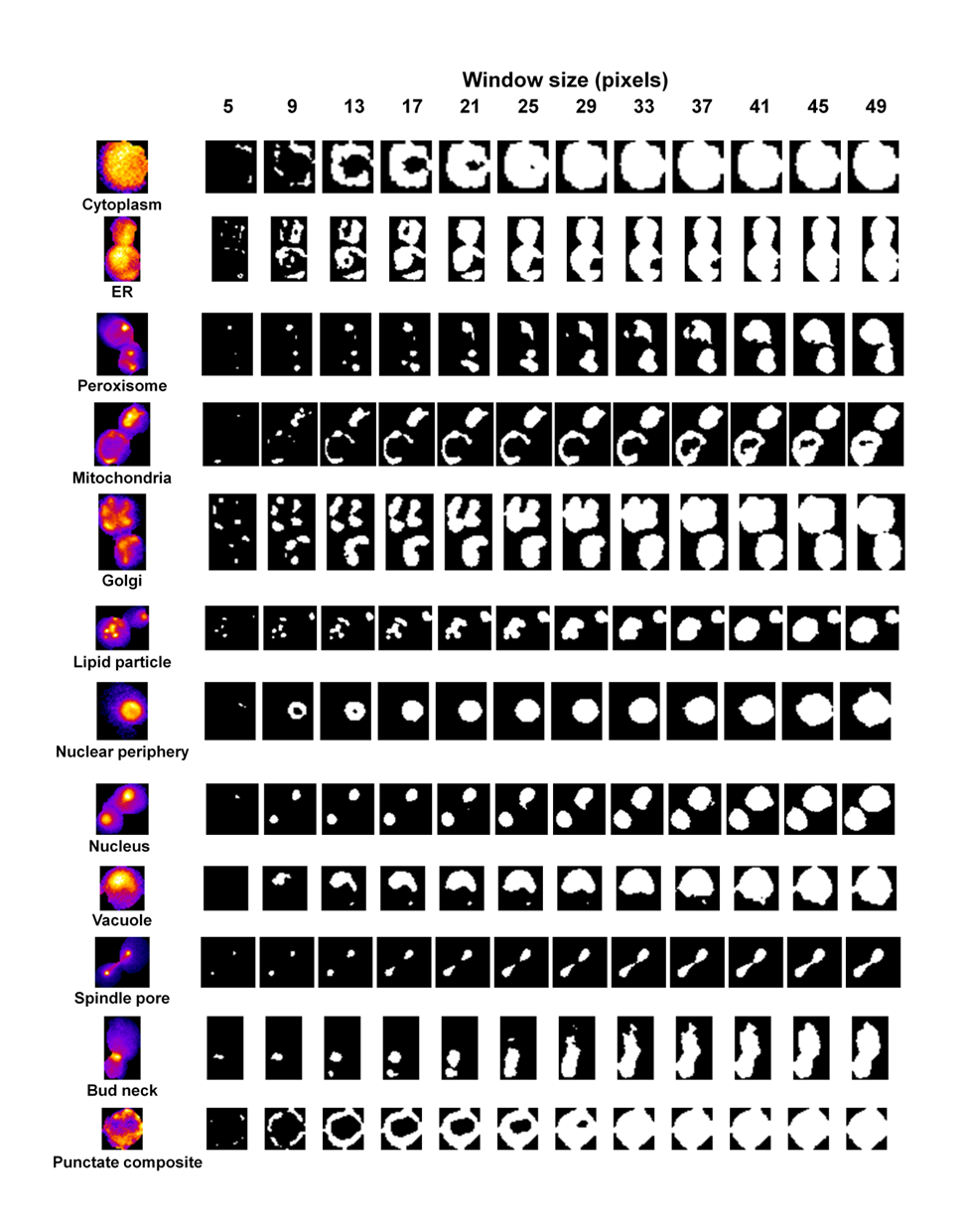

Supplement: Figure S12 — Example of binary local structures detected using different window sizes. (TIF) [file pcbi.1003504.s015.tif]
