# Supplementary material for: Quantitative Protein Localization Signatures Reveal an Association between Spatial and Functional Divergences of Proteins
Source: PLoS Comput Biol. 2014 Mar 6;10(3):e1003504. doi: 10.1371/journal.pcbi.1003504 (PMC3945119; doi:10.1371/journal.pcbi.1003504)
Supplement: Text S1 — Sources of the datasets that we used in our study. (DOCX) [file pcbi.1003504.s016.docx]

## Data sources

| Dataset (version) | Institute | Reference | Source | Note |
| --- | --- | --- | --- | --- |
| Yeast GFP image dataset and annotations | University of California, San Francisco (UCSF) | Huh, et al., Nature, 2003 | <http://yeastgfp.ucsf.edu> | The original website is no longer online, but a copy of the image dataset is still available at the Yeast Resource Center (<http://www.yeastrc.org/pdr/pages/download.jsp>) |
| Gene Ontology annotation (org.Sc.sgd.db, v2.9.1) | Saccharomyces Genome Database (SGD) | - | [http://www.bioconductor.org/packages/2.13/data/annotation/html/org.Sc.sgd.db.html](http://www.bioconductor.org/packages/2.11/data/annotation/html/org.Sc.sgd.db.html) | - |
| Valid ORFs (last modified 3 Feb 2011) | Saccharomyces Genome Database (SGD) | - | <http://downloads.yeastgenome.org/sequence/S288C_reference/orf_protein/orf_trans.fasta.gz> | Except "Dubious" ORFs and pseudogenes |
| GO Slim BP, MF, and CC categories (downloaded 15 June 2013) | Saccharomyces Genome Database (SGD) | - | <http://downloads.yeastgenome.org/curation/literature/go_slim_mapping.tab> | We did not consider ORFs assigned to the "other" category and "IEA" evidence code |
| GO protein complexes (downloaded 15 June 2013) | Saccharomyces Genome Database (SGD) | - | <http://downloads.yeastgenome.org/curation/literature/go_protein_complex_slim.tab> | Only complexes with 5 or more subunits were used |
| Affinity-purification mass spectrometry and yeast two-hybrid protein interaction dataset | Dana-Farber Cancer Institute | Yu, et al., Science, 2008 | <http://interactome.dfci.harvard.edu/S_cerevisiae/index.php?page=download> | We used the Combined-AP/MS and Y2H-union datasets |
| Orthologous gene sets for Ascomycota fungi | Broad Institute | Wapinski, et al., Nature, 2007 | <http://www.broadinstitute.org/regev/orthogroups/> | We used all-output.txt from the website |
| WGD duplicate gene set | Smurfit Institute of Genetics | Gordon, et al., PLOS Genetics, 2009 | <http://dx.doi.org/10.1371/journal.pgen.1000485> | Table S1 |
